# Raising the bar: a systems approach to promoting good wellbeing and self-efficacy in zoo and aquarium professionals

**DOI:** 10.3389/fvets.2026.1746998

**Published:** 2026-07-09

**Authors:** Sabrina Brando, Hannah M. Buchanan-Smith, Sonia Rey Planellas, Line Caes

**Affiliations:** 1 InterBeing AnimalConcepts, Alicante, Spain; 2 Department of Psychology, Faculty of Natural Sciences, University of Stirling, Stirling, United Kingdom; 3 IRTA (Institute of Agrifood Research and Technology), Animal Welfare Program, Girona, Spain

**Keywords:** aquariums, employee wellbeing, leadership, learned helplessness, self-efficacy, working conditions, zoos

## Abstract

This research examines life satisfaction, individual and organisational job satisfaction, and self-efficacy in zoo and aquarium (ZOAQ) professionals, investigating how organisational structures, processes, provisions, and team engagement contribute to these outcomes. We applied a novel integration of Bronfenbrenner’s Ecological Systems Theory, the Rhinelands Way, and the One Welfare framework to guide a mixed-methods study comprising a cross-sectional mixed-methods survey (*n* = 442) and follow-up interviews (*n* = 39) across 23 accredited organisations. Descriptive statistics, correlations, mediation and moderation analyses addressed five pre-specified hypotheses, with thematic analysis of interview data contextualising the quantitative findings. Most participants reported satisfactory individual job satisfaction (71%), and work perceptions and organisational appreciation were more strongly associated with job satisfaction than access to tangible provisions. However, findings revealed significant role-based disparities in provision access, a weak relationship between formal wellbeing processes and job satisfaction, and patterns of manager-observed behaviour and self-efficacy suppression consistent with learned helplessness. These results provide an empirical basis for developing people-centred organisational cultures characterised by equitable provisions, supportive leadership, meaningful communication, and structures that promote help-seeking and collective goals including animal wellbeing and conservation. Limitations include the cross-sectional design, English-language sampling, and the inferential rather than direct measurement of learned helplessness.

## 1 Introduction

Employee wellbeing in zoos and aquariums (henceforth ZOAQs) is gaining attention because of the negative effects of poor wellbeing on individuals and organisations (1). Although animal care workers find their profession meaningful (1, 2), they report concerns about health, burnout, and job stressors (1, 3–6). These issues can lead to high turnover, mistakes, reduced productivity, learned helplessness, and poor job satisfaction (1, 7–10). Understanding how to promote and sustain good employee wellbeing in ZOAQs has become a shared objective for leadership teams, professional associations (including WAZA, AZA, and ZAA), and researchers working at the intersection of occupational wellbeing in animal and conservation professions.

This research addresses a wide range of interconnected factors of wellbeing, including satisfaction with life, individual and organisational job satisfaction, self-efficacy, and explores these by examining the individual, team, leadership, and organisational aspects of employee wellbeing. Although there is no unified definition of wellbeing (11, 12), it is commonly defined as “how people feel and function both personally and socially, and how they evaluate their lives” [(13), p. 6]. Wellbeing reflects fulfilment across life domains, including family, social relationships, work, and the environment (14, 15). While job satisfaction contributes to occupational wellbeing (16), factors like long hours and poor work-life balance (17) can reduce life satisfaction (18).

Satisfaction with life is a component of wellbeing that reflects how meaningful individuals perceive their lives to be (19). It is linked to psychological wellbeing and subjective wellbeing, which are distinct yet related concepts (20, 21). Subjective wellbeing involves cognitive appraisal of life satisfaction and emotional stability (22–24). In contrast, psychological wellbeing encompasses flourishing, good health, autonomy, personal development, relationships, purpose, and environmental mastery (20, 25). While subjective wellbeing is associated with happiness, psychological wellbeing focuses on realising an individual’s potential and building resilience. Improving job quality can enhance life satisfaction, as (18, 26) identified a reciprocal relationship between life and job satisfaction. In the context of ZOAQs, where the nature of the work itself is a source of meaning, both forms of wellbeing are relevant: subjective wellbeing captures how employees feel about their overall life, while psychological wellbeing captures whether they experience autonomy, growth, and purpose in their role - conditions that the present study examines through individual and organisational job satisfaction, self-efficacy, and life satisfaction.

Job satisfaction is a “pleasurable or positive emotional state, resulting from the appraisal of one’s job experiences” [(27), p. 316]. It includes individual satisfaction, measured by overall work contentment, and organisational satisfaction, assessed through support, appreciation, and employee wellbeing. Job satisfaction is influenced by organisational culture, work-life integration, recognition, and professional development (28). Dreer's (29) survey showed that positive workplace emotions strongly predict job satisfaction and retention. Individuals with positive emotions can set goals, maintain expectations, and overcome challenges (30).

Self-efficacy refers to an individual’s belief in their ability to overcome challenges and perform tasks successfully (31), which is “the foundation of human inspiration, motivation, performance accomplishments, and emotional well-being” (32). Self-efficacy is directly related to autonomy and competence satisfaction (33). Studies have shown that workplace self-efficacy correlates with life satisfaction among various professionals (34–36). Self-efficacy also mediates a robust relationship between servant leadership and employee wellbeing, highlighting the importance of supportive leadership traits such as humility, servanthood, and integrity (37).

Appropriate self-efficacy is crucial in caring job positions as repeated exposure to uncontrollable stressors and events, such as inefficient processes, constant criticism, toxic work culture, overwork, lack of control, and perceptions of injustice, can lead to learned helplessness (8, 38, 39). Learned helplessness is a condition that develops when individuals are repeatedly exposed to aversive or uncontrollable events and come to expect that their responses cannot influence outcomes - an expectation that persists even when control subsequently becomes available (40–42). In occupational settings, stressors such as inefficient processes, persistent criticism, lack of autonomy, and perceived injustice can produce this response (8, 38, 39), with consequences for motivation, engagement, and organisational commitment (43–45). It is important to note that in the present study, learned helplessness was not directly measured via a validated instrument but is inferred from a pattern of convergent evidence, as detailed in the discussion. This distinction is made explicit throughout.

Considering employees’ wellbeing holistically, including general health and safety in organisations, is crucial for maintaining a productive and sustainable work environment. Beyond physical safety measures, psychological wellbeing has gained recognition as a vital component of employee wellbeing (46). Organisations that prioritise psychological wellbeing and subjective wellbeing foster a positive workplace culture, reduce stress, and enhance employee engagement, creating a resilient workforce, reducing absenteeism, and promoting long-term employee retention (10, 37, 47).

Addressing the effects on life and job satisfaction and supporting wellbeing and growth opportunities require a comprehensive strategy throughout the organisation. Achieving collective goals in ZOAQs requires more than resource allocation, it requires leadership that understands employee wellbeing as a relational and ethical commitment, not a transactional one. To understands needs, models healthy practices, and builds enabling structures. This distinction is central to what the first author terms the We-Care approach (47, 48): a proactive framework in which leadership actively participates in a collective care system, attends to individual needs as a prerequisite for shared goals, models the caring culture it seeks to build, and promotes conditions in which people can flourish, rather than simply providing tools for others to achieve outcomes. We-Care is the conceptual foundation from which the One Care model proposed in this paper develops. With missions of good animal wellbeing, conservation of species and habitats, education, and other goals in ZOAQs, human wellbeing is central to achieving these collective goals. The triple bottom line framework (49, 50) encompassing profit, people, and planet. When considering sustainable practices, this framework can be an inspiring pathway to impact society and the environment, alongside financial performance. The “three P’s”: profit, people, and planet conceptualise environmental responsibility, promote practices that positively impact society, and review business practices, including supply chains, partners, and renewable energy use. Care for employee wellbeing is key to sustainable success, where profits serve the common good, aligned with the commitment to care for people, other animals, the community of life, and the planet we share through the lens of InterBeing (51–53), a philosophical framework positing the interconnectedness of all beings and the ethical obligations this entails.

To gain a comprehensive understanding of what contributes to these various aspects of employee wellbeing, this research was guided by three theoretical models: Bronfenbrenner’s ecological systems theory, The Rhinelands Way (henceforth Rhinelands), and the One Welfare approach. The core theory onto which the other two are mapped is Bronfenbrenner’s ecological systems theory..

### 1.1 Bronfenbrenner’s ecological systems theory

Bronfenbrenner’s ecological systems theory (54) (henceforth BronfenbrennerEST), as a foundational framework in developmental psychology, empowers this research through its interconnected and systemic approach, featuring various environmental systems while placing the individual at its centre (55). The theory posits that interconnected environmental systems, ranging from immediate surroundings (e.g., family, work, and peer groups) to broader societal structures such as social norms, culture, and economic systems, influence an individual’s development and psychological growth (56). Specifically, five systems exist: microsystem, mesosystem, exosystem, macrosystem, and chronosystem (Table 1).

**Table 1:** Overview of Bronfenbrenner’s ecological systems.

System	Description
The microsystem (M1)	Focusses on the immediate environment of the individual including family, friends, school, work, and the neighbourhood.
The mesosystem (M2)	Focusses on the connections between the different microsystems, such as the relationship between home and the neighbourhood, home and school, or work and home.
The exosystem (M3)	Focusses on the external environments such as the workplace or media influence.
The macrosystem (M4)	Includes broader influences such as the economic conditions, cultural values, traditions, and political ideologies.
The chronosystem (M5)	Encompasses the dimension of time and changes that occur throughout life, such as life transitions and historical events, impact and influence development.

Together, all five systems provide an integrated approach that illuminates individual, team, leadership, and organisational aspects affecting employee wellbeing. While emphasising the dynamic interplay between individual and environment, each system plays a distinct role in developing proximal and distal influences.

Proximal and distal influences are essential in BronfenbrennerEST. Proximal influences involve direct interactions with surroundings, such as relationships with family, peers, co-workers, and leadership. These include physical environment, activities, and routines, which strongly impact wellbeing and are more observable. Examples in ZOAQs include team coherence and interactions with animals. Distal influences include societal factors like socioeconomic status, traditions, cultural norms, values towards ZOAQs, and political systems such as government education policies. Distal influences have indirect, longer-term effects on wellbeing and are harder to measure.

While BronfenbrennerEST has been used mainly in child development, it has found broader application in understanding complex systems. It has been successfully used in work-related stress interventions (57), education (58, 59), workplace health promotion (60), community development (61), employability (62), guiding public mental health policy (63), campus ecology (64), and creating inclusive educational environments (65). ZOAQ is an ecosystem of systems with levels from frontline employees to external stakeholders like visitors and communities. The Bronfenbrenner model provides a holistic approach to understand and improve ZOAQ’s employee wellbeing. The M1 system reveals policies, processes, opportunities like CPD, volunteering, and provisions that promote wellbeing. The M2 system shows collaboration, communication, and organizational structure effects. M3 demonstrates how opportunities affect employee wellbeing, whereas M4 reveals broader cultural influences. M5’s time lens and system interactions drive holistic staff wellbeing programs supporting collective goals.

### 1.2 Rhinelands

The Rhinelands approach emphasises stakeholder-oriented business management, contrasting the shareholder-centric Anglo-Saxon model (66). The term Anglo-Saxon here refers to a model of corporate governance, not a geographic or cultural designation, and adherents of both philosophies are found across Europe, North America, and beyond (67). The Rhineland philosophy prioritises sustainability and considers interests of employees, customers, suppliers, and community. Companies build relationships, foster innovation, and maintain social responsibility through stakeholder consultations. The approach features labour unions, training programs, and quality expertise. While sacrificing short-term profits, it creates stability and long-term success (68).

### 1.3 One Welfare

The One Welfare approach recognises the interconnectedness of animal welfare, human wellbeing, and environmental sustainability (69). This emphasises that improving one aspect impacts the others positively. This holistic framework promotes collaboration across disciplines to address the complex challenges of animal care, public health and environmental conservation. By considering these interconnected elements, One Welfare aims to create sustainable solutions that benefit animals, humans, and the planet (70).

Using Bronfenbrenner’s theory, combined with an all-stakeholder organisational structure (Rhinelands) and an interconnected human-animal-environment approach (One Welfare), we adopted a systems approach to explore the aspects that influence individual wellbeing of ZOAQs at the individual, team, leadership, and organisational levels.

Throughout the article, italics denote variables, while these words used elsewhere non-italicised do not reflect variables in this study. This study examines which aspects of ZOAQ employees’ roles across organisational levels, such as holistic wellbeing processes (henceforth *holprocesses*)*, provisions, and opportunities,* support their *life satisfaction*, Individual job satisfaction (henceforth *Indjobsat*), Organisational job satisfaction (henceforth *Orgjobsat*), and *self-efficacy*. Questions related to the Rhineland approach are labelled as Rhineland individual (henceforth *RHInd*) and Rhineland organisational (henceforth *RHOrg*). We included employees from different organisational levels to provide a broader perspective and tested hypotheses across Team and Managers levels and Organisational support and structure level. Specifically, we tested five hypotheses.

### 1.4 Integrating the frameworks

The combined lens of these three frameworks makes it possible to examine employee wellbeing in ZOAQs not as a property of individuals, but as something produced - and reproducible, at the intersection of structural conditions, organisational philosophy, and relational ethics. BronfenbrennerEST provides the structural scaffolding - mapping how individual, team, leadership, and organisational factors nest within and interact across levels. The Rhinelands approach brings a practical and organisational philosophy that centres multi-stakeholder engagement and long-term sustainability over short-term extraction. One Welfare situates human wellbeing within the broader relational context of animal welfare and environmental sustainability. What this means in practice is that understanding why a frontline animal caregiver feels competent, satisfied, or depleted requires attending simultaneously to their immediate working conditions, the management culture they operate within, the organisational systems that support or constrain them, and the broader sector values that define what their work is for. Together, these frameworks make possible a study of employee wellbeing that is simultaneously individual, institutional, ethical, and ecological in scope.

Based on this integrated approach, this study aimed to examine how individual, team, and organisational factors contribute to life satisfaction, individual and organisational job satisfaction, and self-efficacy in ZOAQ professionals across five pre-specified hypotheses to test specific aspects of this multi-level picture. The hypotheses are organised across two levels of analysis reflecting the structure of the survey: the team and managerial level, examining how day-to-day working conditions and engagement shape individual outcomes; and the organisational support and structure level, examining how broader processes and leadership philosophy contribute to wellbeing. Together they operationalise the systems approach described above into testable relationships.

#### 1.4.1 Team and managers

The following four hypotheses were tested at both the team and manager levels, depending on the whether the survey question was completed by the full sample or managers only. At the team level, the dataset included all participants across all ZOAQs levels. At the managerial level, participants who identified as managers (staff members supervising others) answered additional questions related to specific working conditions.

1. Individuals reporting good working conditions (*provisions/opportunities*) report higher levels of *Indjobsat*, *Orgjobsat* and *life satisfaction.*
2. Levels of *self-efficacy* and *experience* mediates/moderates* the relationship between good working conditions (*provisions/opportunities*) and *Indjobsat*, *Orgjobsat* and *life satisfaction*.
3. Individuals reporting good *individual* and *team* engagement report higher levels of *self-efficacy, Indjobsat, Orgjobsat* and *life satisfaction*.
4. Levels of *self-efficacy* and *experience* mediates/moderates* the relationship between *team* engagement, *Indjobsat, Orgjobsat,* and *life satisfaction*.

*Mediating variables clarify the process by which an independent variable influences the outcome, detailing the underlying pathways. In contrast, moderating variables affect the intensity or direction of the relationship between two variables.

*Organisational support and structure*: Organisational support- and structure-related questions were answered only by managers.

1. Managers reporting more holistic employee wellbeing processes (*holprocesses*) report higher levels of *Indjobsat* and *Orgjobsat* and *life satisfaction*.

## 2 Materials and methods

This project employed a cross-sectional mixed-methods design, approved by the General University Ethics Panel (GUEP) at the University of Stirling, Scotland, with reference number GUEP 2023 711,811,265. Quantitative surveys and qualitative interviews were used to gain insights into the individual, team, leadership, and organisational aspects of employee wellbeing in ZOAQs. This study was designed for online data collection. The web-based survey was administered through University of Stirling’ Qualtrics licence. Online interviews were conducted using the University’s Microsoft Teams licence. The survey ran from 11/07/2023 to 28/09/2023. Interviews were conducted after the survey closed between 26/10/2023 and 30/01/2024.

Olmos-Vega et al. (71) frame reflexivity as a productive engagement with researcher subjectivity rather than its elimination. Lead author (SB) believes reflexivity should applied to all studies. SB coded all interview transcripts and led the quantitative analyses. To mitigate the risks associated with single-researcher coding, the following practices were employed: (1) Two coding rounds were conducted in NVIVO 1.7.1, with a minimum 1-month interval between rounds. (2) Emerging codes and themes were shared with co-authors (HBS, LC, SRP) at three structured points during the analysis - following initial coding, after thematic consolidation, and at draft Discussion stage. Co-authors challenged interpretations that appeared to go beyond the data, and all such challenges were documented and resolved prior to finalisation. (3) A deductive anchor was maintained: the five pre-specified hypotheses constrained interpretive scope and provided a reference framework against which qualitative themes were evaluated. (4) Quotes were selected to illustrate themes, not to build arguments from scratch - both confirmatory and disconfirmatory accounts are represented in the discussion, reflect demographic diversity across job roles, and represent views expressed by multiple participants rather than outliers. Quotes contextualise findings in the discussion section, as advised by (72), and highlighted by Byrne (73) when discussing reporting styles. Unless otherwise stated, all quotes are from this study. SB has worked in ZOAQs for over 37 years and is the founder of InterBeing AnimalConcepts, a consultancy in this domain. This professional experience informed the research design and provided ecological validity; it was counterbalanced by academic supervision at the University of Stirling and formal ethics oversight (GUEP 2023 711,811,265).

### 2.1 Participants

#### 2.1.1 Recruitment

The initial list of candidate organisations was compiled from published membership directories of four accreditation bodies: the Association of Zoos and Aquariums (AZA), the Zoo and Aquarium Association (ZAA), the European Association of Zoos and Aquariums (EAZA), and the British and Irish Association of Zoos and Aquariums (BIAZA). Organisations were screened for English-language operation and a minimum of 50 staff using publicly available information such as on Linkedin. A senior contact was identified through publicly available institutional information, and use of network contacts. Each organisation received a personalised invitation email from the lead author (SB), with a single follow-up email sent 14 days later if no response was received. No telephone contact was made. Of the 52 organisations approached, 23 accepted, 16 declined, and 13 did not respond. Organisations across seven countries participated: the United Kingdom (1), the United States (7), Canada (3), Australia (8), New Zealand (2), Singapore (1), and China (1).

#### 2.1.2 Roles of participants

A purposive sampling method identified participants based on their organisational role. Each organisation received a letter with a survey link and list of identified roles. While recognising the variety of roles in ZOAQs, this study focused on those directly involved in animal care and wellbeing. Participants were recruited across these roles: Junior animal caregiver (Junior), Senior animal caregiver (Senior), Curator, Veterinarian, Veterinary professional (e.g., veterinary nurse), Animal welfare scientist/coordinator (AWS), CEO, and Other (e.g., nutritionists, registrars, directors).

#### 2.1.3 Sample size determination

Owing to the originality of this research and the lack of data on effect size, a power calculation for the sample size was not possible. Our proposed sample size of 250–300 participants was based on obtaining adequate representation across job/career-level positions and capturing variability across ZOAQs worldwide. Previous research on laboratory animal technicians [(74) *n* = 90], animal care workers [(75) *n* = 139], and veterinary teams [(76) *n* = 274] found this sample size sufficient for statistically meaningful results. We requested approximately 25 staff members per facility to complete an online survey. The target interview sample size was set at minimum 25, approximately 10% of desired sample size, to reach saturation, defined as information redundancy by Lincoln and Guba (77). However, recent developments suggest sample size cannot be determined before data collection or analysis (78). The data could include thinner or shorter individual items, or text from interviews (78). Following Clark et al. (79), the first author evaluated data during interviews to assess quality, richness, and meaning diversity to determine sufficient interviews. Of 483 survey participants, 442 completed at least half, with 91.50% completion rate. Of these, 415 completed the survey. Data were included if more than half was completed. In the second part, 113 self-identified managers completed this section, including all positions except Junior animal caregiver. Sample sizes vary across analyses as not all participants responded to every question; exact n is reported for each analysis. The sample is broadly representative of frontline and mid-level ZOAQ roles in accredited, English-speaking institutions. Leadership roles are underrepresented. No published data on the exact distribution of roles within large ZOAQs exist to enable formal representativeness testing.

#### 2.1.4 Survey and interview design

The Qualtrics platform was configured to prevent multiple submissions from the same browser session. As the survey was distributed via organisational contacts rather than publicly, duplicate participation was further limited by the targeted distribution method. The survey comprised two parts: Part 1 completed by all participants (assessing life satisfaction, self-efficacy, individual job satisfaction, organisational job satisfaction, individual and team engagement, provisions, opportunities, and Rhinelands individual items); Part 2 was completed by managers only (assessing manager observations, working conditions, Rhinelands organisational items, and holistic wellbeing processes). The survey consisted of approximately 80 items across all sections, with a Prefer not to say option added to all questions. The survey was piloted with ZOAQ professionals who could provide feedback and comments.

See below for the details of the relevant survey questions for the analysis in this article. It is important to note that the provisions and opportunities checklists are researcher-developed instruments informed by existing literature on workplace resources in animal care professions (1, 76, 80) and practitioner knowledge. They have not been formally psychometrically validated as scales; accordingly, the provision and opportunity totals are treated as descriptive counts rather than latent construct measures. Readers should interpret correlations involving these variables with appropriate caution.

Identifying information from the survey or interviews was removed to anonymise profiles. Participants were assigned unique identifiers; no cases of apparent duplication were identified in data screening. All participants provided written informed consent prior to survey completion. Participation was voluntary and participants could withdraw at any point. Data are stored on University of Stirling secure servers in accordance with GDPR. Participating organisations received a briefing document outlining the study purpose and participant roles prior to survey launch. No interviewer training was conducted as all interviews were led by the same researcher (SB). Interview questions were developed by the research team and reviewed by co-authors prior to use. Semi-structured interviews were conducted with 39 participants (20 male, 19 female) who had completed the survey and volunteered for follow-up. Interviews were conducted online via Microsoft Teams between 26 October 2023 and 30 January 2024, lasting approximately 1 h each. Questions focused on participants’ lived experiences of their working conditions, organisational support, and wellbeing - for example, ‘Share how you feel supported in your job by your organisation’ and ‘Share how you feel supported at work to care for your own wellbeing.’ All interviews were recorded, transcribed, and anonymised, with participants assigned unique identification numbers. Thematic analysis following Braun and Clarke (78) was used, including data familiarisation, code generation across two rounds in NVIVO 1.7.1 with a minimum two-week interval between rounds, and thematic consolidation reviewed with co-authors. Quotes are used throughout the Discussion to contextualise and illustrate quantitative findings, selected to reflect demographic diversity across roles and the range of views expressed. All relevant survey and interview questions are provided in the Supplementary materials.

##### 2.1.4.1 Questions completed by ALL participants

Life satisfaction was assessed using the Satisfaction with Life Scale (22). This 5-item, 7-point Likert scale assesses global cognitive judgments (81). Participants rated each item on an adapted 5-point scale ranging from 1 (strongly disagree) to 5 (strongly agree). This adaptation maintained consistent response options across all Likert-scale questions in the survey. The total score was calculated by adding all item scores. The adapted version had a Cronbach’s alpha of 0.89 (0.87 in (22)). General self-efficacy was assessed using the PROMIS^®^ Item Bank v.1.0—General Self-Efficacy, April 2020 version (82), which isa 10-item scale with 5-point Likert responses. Participants rated their confidence on each item from 1 = “I am not at all confident” to 5 = “I am very confident.” The total score was calculated by adding all items. This scale is valid and reliable (83), with a Cronbach’s alpha of 0.93 in our sample.

The validated single-item job satisfaction question, *On the whole, how satisfied are you with your job?* (84) used a 5-point scale from 1 (strongly disagree) to 5 (strongly agree).

The organisational job satisfaction questions were adapted from Boivin and Markert (80). It included 5/6 original questions, all positively worded. Questions were assessed using a 5-point Likert scale (adapted from a 6-point scale), from 1 = “strongly disagree” to 5 = “strongly agree.” Cronbach’s alpha was 0.93, and we calculated the mean score for analysis.

For individual and team engagement, questions from Moore et al. (76) were used, where participants indicated agreement with items (five for individual and five for team engagement) using a 5-point scale from = “strongly disagree” to 5 = “strongly agree.” The Cronbach’s alpha was.79 for team engagement [0.89 in (76)] and 0.79 for individual engagement [0.89 in (76)]. The mean score was calculated for analysis, with higher scores reflecting stronger engagement.

For provisions that employees have access to (healthcare, appropriate clothing, opportunities for reward), participants answered *Yes, No, or Prefer not to say* to 12 different relevant provisions. A total score was created ranging from 0–12.

For the seven questions regarding employee access to opportunities like courses, financial training support, and workday events, response options were *Yes, No, or Prefer not to say.* A total score was created, ranging from 0–7. For Q1-Q6, Yes was scored 1 and No was scored 0, reflecting presence of access. Q7 (‘I have no access to continued professional development’) was reverse scored (No = 1, Yes = 0), consistent with the directional logic of the scale, such that higher total scores reflect greater overall access to CPD opportunities across all seven items.

It should be noted that the provisions and opportunities checklists are researcher-developed descriptive inventories informed by existing literature on workplace wellbeing resources in animal care professions (1, 76) and practitioner knowledge of ZOAQ working conditions. They have not been formally validated as psychometric scales; accordingly, the provision and opportunity totals are treated as descriptive counts rather than latent construct measures, and correlations involving these variables should be interpreted with appropriate caution.

For the six questions related to Rhinelands at the individual level (*RHInd*), participants rated their agreement with items using a 5-point scale from 1 = “strongly disagree” to 5 = “strongly agree.” The Cronbach Alpha was 0.80. A mean score of six items was calculated, with higher scores reflecting greater autonomy, feeling supported and trusted by the organisation, and being proud of one’s work.

##### 2.1.4.2 Questions completed by participants identifying themselves as managers

To assess working conditions from a managerial perspective, 15 negatively valanced questions were used to gauge what managers observe in their teams, such as reduced desire to work with people, reduced problem-solving capacity, or neglecting personal wellbeing needs. The questions used a 5-point scale from 1 = “strongly disagree” to 5 = “strongly agree.” A sum score of the 15 questions was calculated for analyses, with higher scores representing more reported observations.

To assess working conditions with their teams and management, 5 questions were used, such as I feeling trapped or supported. The questions used a 5-point scale from 1 = “strongly disagree” to 5 = “strongly agree.”

For the 16 Rhinelands questions at the organizational level (*RHOrg*), managers indicated their agreement with each item using a 5-point scale ranging from 1 = “strongly disagree” to 5 = “strongly agree.” The Cronbach Alpha was 0.66. A mean score was calculated, with higher scores reflecting a greater focus on collaboration, collective goals, and trust in expertise.

To asses holistic employee wellbeing processes (*holprocesses*), a series of ISO certification questions from 45,001 and 45,003 standards, including risk assessment, back-to-work programs, communication, consultation, whistleblower policy, and audits, were assessed using a 5-point scale, from 1 = “strongly disagree” to 5 = “strongly agree.”

### 2.2 Statistical analysis

Given that most variables departed significantly from normality (confirmed by Shapiro–Wilk tests), non-parametric Spearman rank correlations were used throughout for bivariate hypothesis testing. Mediation and moderation analyses were conducted using Hayes’ (130) PROCESS macro (Model 4 for mediation, Model 1 for moderation), which employs bootstrapped confidence intervals and is appropriate for non-normally distributed mediators and moderators. Bootstrapping used 5,000 samples at a 95% confidence interval. All mediation analyses tested direct effects first; indirect effects were only examined where direct effects were significant.

Statistical analyses were conducted using SPSS version 28.0.0.0 (190). Descriptive analysis (frequencies, means, median, standard deviation) was used for demographics including gender, age, job position, time in the field (henceforth *experience*), time working, supervision, union, courses, and others in similar positions. Crosstab contingency tables examined relationships between job position, financial compensation, *self-efficacy, experience, life satisfaction, Indjobsat, Orgjobsat, team* and *individual* engagement, *RHInd, sum of processes, holprocesses, provisions,* and *opportunities*. No imputation or transformation of variables was performed. All scales are used in their original scored form with adaptations described above.

**Figure 1:**
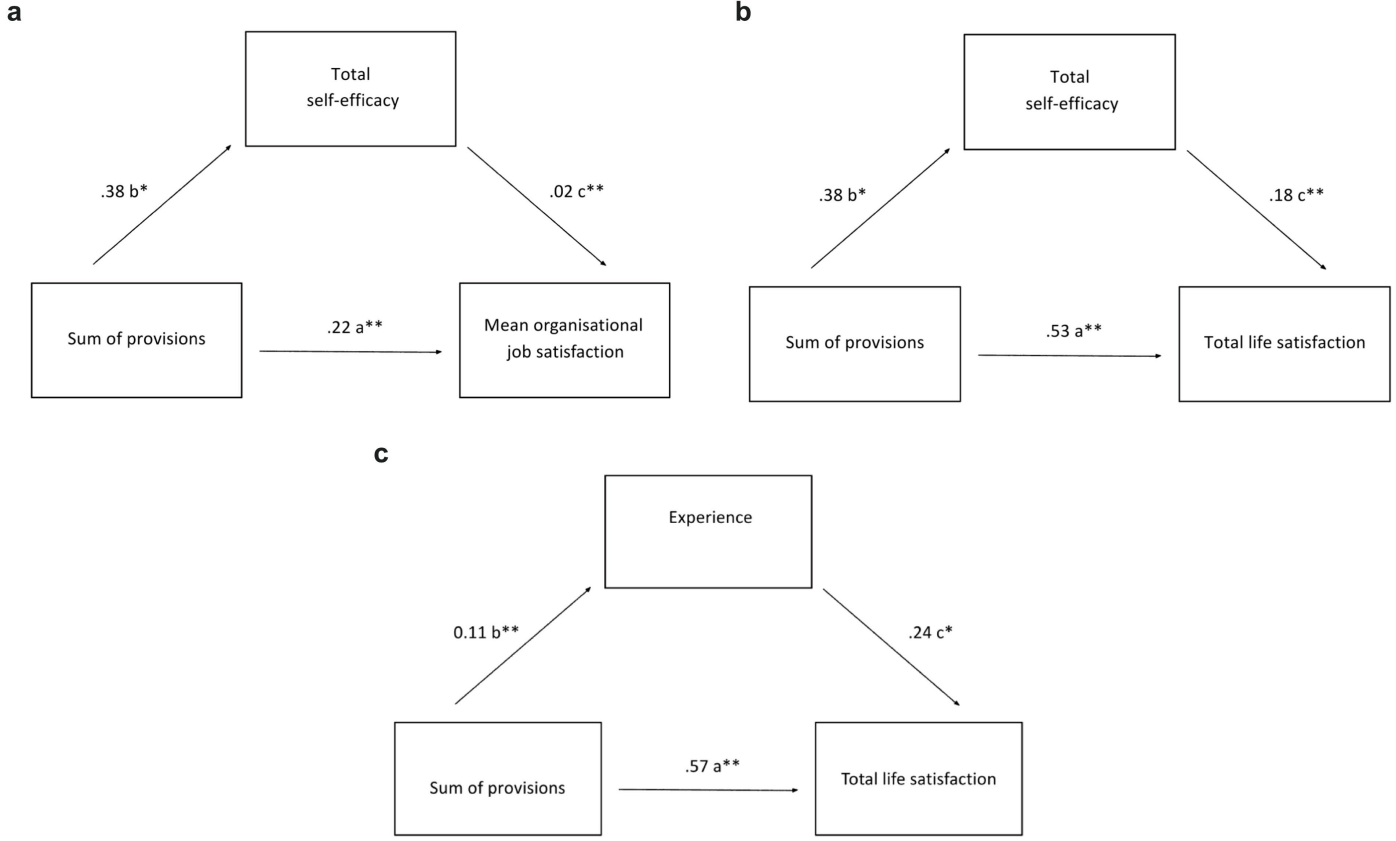
**(a)** Self-efficacy, sum of provisions, and mean organisational job satisfaction. This figure illustrates the mediation model for the sum of provisions, mean *Orgjobsat*, and total *self-efficacy*. * = *p* < 0.05 ** = *p* < 0.001. **(b)** Self-efficacy, sum of provisions, and total life satisfaction. This figure illustrates the mediation model for the sum of provisions, individual job satisfaction, and total self-efficacy. * = *p* < 0.05 ** = *p* < 0.001. **(c)** Experience, Sum of Provisions, and Total Life Satisfaction. This figure illustrates the mediation model for the sum of provisions, total life satisfaction, and experience. * = *p*< 0.05 ** = *p*<0.001.

For hypotheses one, three and five, Spearman correlation analyses were conducted. For hypotheses two and four, mediation and moderation analyses were conducted with *self-efficacy* and *experience* for outcome variables of *Indjobsat* and *Orgjobsa*t, and *life satisfaction*. For mediation, we tested in SPSS PROCESS macro with model 4 the direct relationship between variables, and if found, further tested the indirect effects via the mediator. For example, in Figure 1, the direct effect between *provisions* and *Indjobsa*t is path A, between *provisions* and *self-efficacy* is path B, and between *self-efficacy* and *job satisfaction* is path C. All mediation figures will be structured and labelled as such.

For moderation, we tested the SPSS PROCESS macro using Model 1, first exploring the direct effects between all relevant variables and then testing the interaction effect between the independent variable and moderator at three levels of the moderator: low, medium, and high.

For the *RHOrg* questions a Principal Component Analyses (PCA; without any rotations) was run to identify the essential aspects and structure the items measuring *RHOrg* represent, given this is the first translation of this approach into questions aligned with the *RHOrg.* To decide on the number of components to retain, the scree plot and Eigenvalues were interpreted simultaneously, retaining any factors with an Eigenvalue over 1. The component matrix was used to identify the item distribution across the retained components, disregarding any items with a factor loading below 0.32. Sample size varies slightly across all analyses as not all participants responded to every question. We included the exact sample size for each question in figures and tables. No weighting or propensity score adjustment was applied. The sample is not population-representative and findings should not be generalised to all ZOAQ professionals. Role-stratified analyses are presented to allow readers to interpret patterns within role groups.

## 3 Results

Throughout the results, italicised terms denote study variables as defined in the introduction, these are holistic wellbeing processes (henceforth *holprocesses*)*, provisions, and opportunities, life satisfaction*, Individual job satisfaction (henceforth *Indjobsat*), Organisational job satisfaction (henceforth *Orgjobsat*), Rhineland individual (henceforth *RHInd*) and Rhineland organisational (henceforth *RHOrg*). Results are organised by hypothesis, with exact sample sizes reported for each analysis given variation in item completion across participants.

### 3.1 Demographics

A total of 442 participants from 23 ZOAQs across seven countries completed at least half the survey (overall completion rate: 91.5%). Most participants were female (*n* = 296). The most frequent role was senior animal caregiver (*n* = 185, 41.9%), followed by junior animal caregiver (*n* = 87, 19.7%). Participants were predominantly in the 31–35 age bracket, with most reporting 6–10 years of experience in the field. A BSc was the most commonly reported highest qualification. Detailed demographic data are presented in Table 2.

**Table 2:** The survey’s demographic questions including job position, gender, age, years working in the field, highest educational level, considering job a calling, helping profession, number of hours working, supervision, and union for all the participants in the dataset.

	*N*	%	*P*	Skew
Gender *N* = 438				
Female	296	67		
Male	137	31		
Non-binary	5	1.1		
Prefer not the say	4	0.9		
Age *N* = 434			<0.001	W(410) = 0.931
21–25	35	7.9		
26–30	80	18.1		
31–35	95	21.5		
36–40	66	14.9		
41–45	43	9.7		
46–50	47	10.6		
51–55	28	6.3		
56–60	27	6.1		
61–65	9	2.0		
66+	4	0.9		
Prefer not to say	8	1.8		
Job position *N* = 437			<0.001	W(410) = 0.760
Junior animal caregiver	87	19.7		
Senior animal caregiver	185	41.9		
Curator	48	10.9		
Veterinary professional, e.g., veterinary nurse	19	4.3		
Veterinarian	15	3.4		
Animal welfare scientist/coordinator	23	5.2		
Other	50	11.3		
CEO	10	2.3		
Prefer not to say	5	11		
Experience (years in the field) *N* = 433			<0.001	W(433) = 0.909
Less than a year	9	2.0		
1–5 years	87	19.7		
6–10 years	98	22.2		
11–15 years	70	15.8		
16–20 years	61	13.8		
21–25 years	44	10.0		
More than 25 years	64	14.5		
Not working with animals	5	1.1		
Prefer not to say	4	0.9		
I work *N* = 442				
Full-time	417	94.3		
Part-time (more than 20 h)	17	3.5		
Part-time (less than 20 h)	8	1.8		
Prefer not to say	5	1.1		
Does your current position include supervision of other employees? *N* = 439				
Yes	272	61.5		
No	167	37.8		
Prefer not to say	3	0.7		
My facility has a union *N* = 435				
Yes	235	53.2		
No	158	35.7		
I do not know	41	9.3		
Prefer not to say	1	0.2		
I am part of a union				
Yes	118	26.7		
No	309	69.9		
I do not know	11	2.5		
Prefer not to say	2	0.5		
(M) I have worked in this managerial position for *N* = 111			*p* < 0.001	W = 0.858
Less than a year	17	15.3		
1–5 years	42	37.8		
6–10 years	22	19.8		
11–15 years	11	9.9		
16–20 years	7	6.3		
21–25 years	7	6.3		
More than 25 years	5	4.5		
(M) I have worked in this facility for *N* = 110			*p* < 0.001	W = 0.858
Less than a year	12	10.9		
1–5 years	27	24.5		
6–10 years	25	22.7		
11–15 years	13	11.8		
16–20 years	11	10		
21–25 years	10	9.1		
More than 25 years	12	4.5		
(M) I started at this facility in the position I am in now. *N* = 112				
Yes	29	25.7		
No, I moved up from another position in this facility	71	62.8		
No, I came from a similar position in another facility	5	4.4		
No, I moved up from another position in another facility	5	4.4		
Prefer not to say	2	1.8		
(M) Over my career I have worked in … number of parks. *N* = 110				
1 park	41	36.3		
2 parks	30	26.5		
3 parks	15	13.3		
4 parks	10	8.8		
5 parks	7	6.2		
6 parks	5	4.4		
10 parks	1	0.9		
22 parks	1	0.9		
(M) Over my career I have worked in … number of states/countries. *N* = 113				
1 state/country	70	61.9		
2 states/countries	15	13.3		
3 states/countries	11	9.7		
4 states/countries	7	6.2		
5 states/countries	6	5.3		
6 states/countries	2	1.8		
8 states/countries	1	0.9		
22 states/countries	1	0.9		
(M) As I moved up the ladder to a leadership position, I have completed people management courses. *N* = 113				
I have completed courses	71	62.8		
I have not completed any courses	38	22.6		
Prefer not to say	4	3.5		
(M) I manage a team. *N* = 113			*p* < 0.001	W = 0.812
1–5 people	23	20.4		
6–10 people	27	23.9		
11–15	16	14.2		
16 or more	43	38.1		
Prefer not to say	4	3.5		
(M) In the organisation there are other managers in a position like mine, e.g., curator, veterinarian. *N* = 113			*p* < 0.001	W = 0.617
I am the only one	70	61.9		
1–5 people	24	21.2		
6–10 people	9	8		
11–15 people	2	1.8		
16–20 people	1	0.9		
21 or more people	7	6.2		

Additional questions for only those who self-identified as managers are labelled with (M) in front cover time working in managerial position, facility, started or moved in a position, moved facility, state, or country, moving up the ladder completed courses, managing a team, and other managers in similar positions. The Shapiro–Wilk Test for Normality (W) was conducted for all variables to understand their distributions.

### 3.2 Hypothesis 1

Hypothesis 1 predicted that individuals reporting better working conditions (provisions, opportunities, and Rhinelands individual scores) would report higher *Indjobsat*, *Orgjobsat*, and *life satisfaction*.

Support for this hypothesis was found, with significant, positive correlations between all working condition variables (i.e., *provisions, opportunities, RHInd*) and all outcome variables (i.e., *RHInd, Orgjobsat* and *life satisfaction*). Spearman correlation analysis (n = 440, includes all participants, including managers, see Table 3) showed a strong positive relationship between *provisions* and *opportunities* with *Orgjobsat* levels and a very strong positive relationship between mean *RHInd* and *Orgjobsat* levels. A moderate positive relationship between *provisions* and *opportunities* and *Indjobsat* levels and a strong positive relationship between mean *RHInd* and *Indjobsat* levels were observed. A weak positive relationship between *provisions* and *opportunities* and *life satisfaction* levels, and a moderate positive relationship between mean *RHInd* and *life satisfaction* were found.

**Table 3:** Spearman rho correlations life satisfaction, *Indjobsat, Orgjobsat*, individual and team engagement, sum of opportunities and provisions, and *RHInd*.

Variables	N	M	Median	SD	1	2	3	4	5	6
1 Mean organisational job satisfaction	440	3.15	3.20	1.16	1.00	0.62*	0.44**	0.42**	0.51**	0.74**
2 Individual job satisfaction	440	3.76	4	1.15		1.00	0.47**	0.37**	0.38**	0.64**
3 Total life satisfaction	440	17.07	17	0.75			1.00	0.29**	0.32**	0.44**
4 Sum opportunities	440	3.13	3	1.61				1.00	0.45**	0.41**
5 Sum provisions	440	5.5	5	2.56					1.00	0.48**
6 Mean Rhinelands Individual	440	3.58	3.66	0.83						1.00

Correlation is significant at the 0.01 level (2-tailed) *p < 0.05, **p < 0.01.

**Figure 2:**
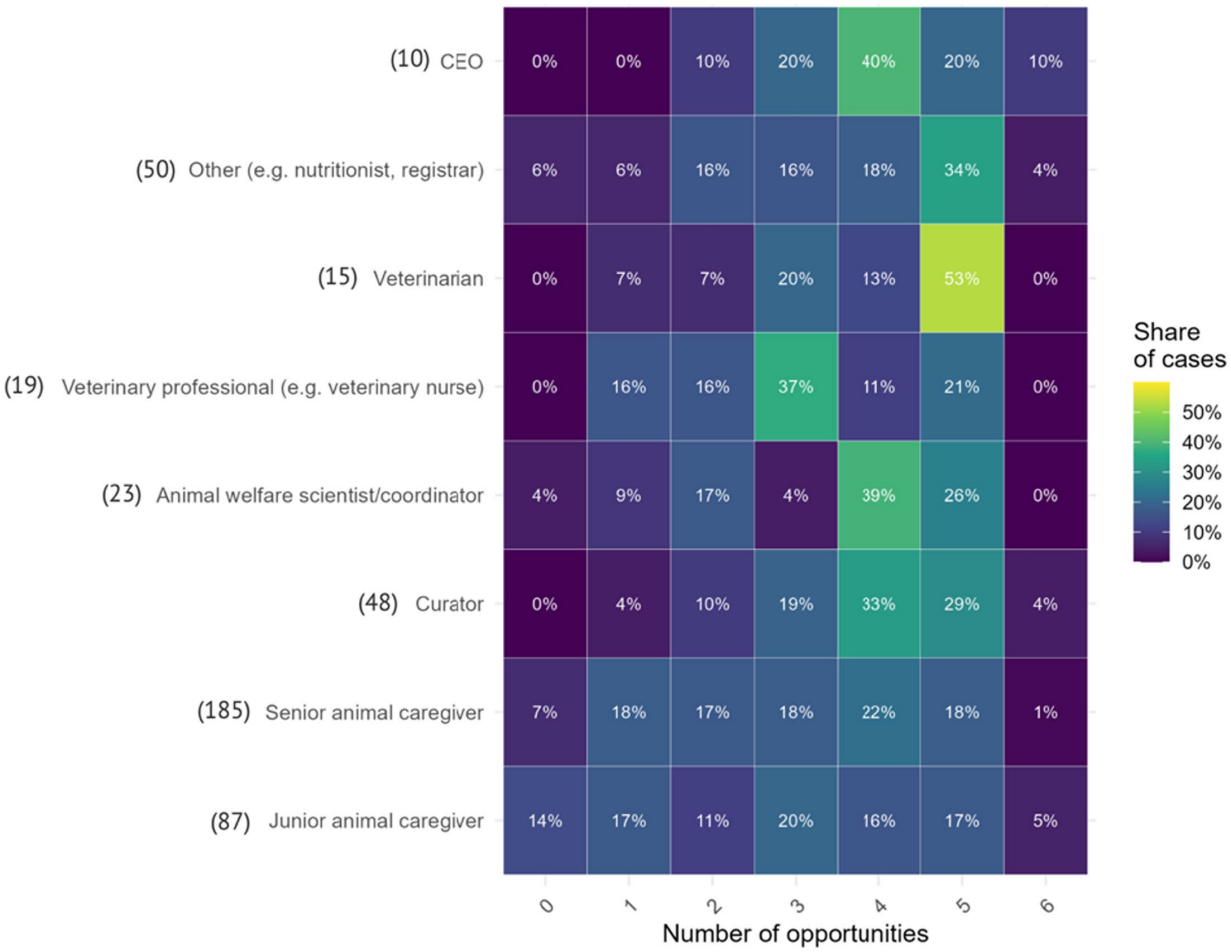
Distribution of opportunities by job position. The heatmap in figure displays the distribution of opportunities by job position, with cells indicating the percentage of opportunities within each role, up to 6 on the *x*-axis. Most veterinarians, CEOs, and individuals in higher positions had four or more opportunities, while most caregivers had three or fewer.

Further on Team level, for the question *Opportunities for development*, most participants (n = 437) responded having continued on-job training (*n* = 269), but less than half indicated satisfactory opportunities for CPD (*n* = 203). For the distribution of opportunities per job role, see all results Table 4 and Figure 2.

**Table 4:** All opportunities for continued professional development per job position.

Question number	Response	Junior (*n* = 87)	Senior (*n* = 185)	Curator (*n* = 48)	AW coordinator (*n* = 23)	Veterinary professional (*n* = 19)	Veterinarian (*n* = 15)	Other (*n* = 50)	CEO (*n* = 10)	Total (*n* = *)
Q1	No	30 (17.9%)	75 (44.6%)	13 (7.7%)	10 (6%)	10 (6%)	5 (3%)	20 (11.9%)	5 (3%)	168 (100%)
	Yes	57 (21.2%)	110 (40.9%)	35 (13%)	13 (4.8%)	9 (3.3%)	10 (3.7%)	30 (11.2%)	5 (1.9%)	269 (100%)
Q2	No	54 (23.1%)	111 (47.4%)	21 (9%)	10 (4.3%)	12 (5.1%)	5 (2.1%)	18 (7.1%)	3 (1.3%)	234 (100%)
	Yes	33 (16.3%)	74 (36.5%)	27 (13.3%)	13 (6.4%)	7 (3.4%)	10 (4.9%)	32 (15.8%)	7 (3.4%)	203 (100%)
Q3	No	59 (22.9%)	106 (41.1%)	25 (8.7%)	13 (4.5%)	16 (5.6%)	9 (3.1%)	29 (10.1%)	6 (2.1%)	287 (100%)
	Yes	28 (15.7%)	78 (43.8%)	23 (15.3%)	10 (6.7%)	3 (2%)	6 (4%)	21 (14%)	4 (2.7%)	150 (100%)
Q4	No	59 (22.9%)	106 (41.1%)	28 (10.9%)	17 (6.6%)	5 (1.9%)	7 (2.7%)	33 (12.8%)	3 (1.2%)	258 (100%)
	Yes	28 (15.7%)	78 (43.8%)	20 (11.2%)	6 (3.4%)	14 (7.9%)	8 (4.5%)	17 (9.6%)	7 (3.9%)	178 (100%)
Q5	No	43 (26.9%)	83 (51.9%)	7 (4.4%)	4 (2.5%)	8 (5%)	2 (1.3%)	12 (7.5%)	1 (0.6%)	160 (100%)
	Yes	44 (15.9%)	102 (36.8%)	41 (14.8%)	19 (6.9%)	11 (4%)	13 (4.7%)	38 (13.7%)	9 (3.2%)	277 (100%)
Q6	No	39 (26.9%)	71 (49%)	9 (6.2%)	5 (3.4%)	5 (3.4%)	2 (1.4%)	12 (8.3%)	2 (1.4%)	145 (100%)
	Yes	48 (16.4%)	114 (39%)	39 (13.4%)	18 (6.2%)	14 (4.8%)	13 (4.5%)	38 (13%)	8 (2.7%)	292 (100%)
Q7	No	11 (36.7%)	14 (46.7%)	0 (0%)	1 (3.3%)	1 (3.3%)	0 (0%)	3 (10%)	0 (0%)	30 (100%)
	Yes	76 (18.7%)	171 (42%)	48 (11.8%)	22 (5.4%)	18 (4.4%)	15 (3.7%)	47 (11.5%)	10 (2.5%)	407 (100%)

Q1. I have continued on the job training, *n* = 437*, Prefer not to say *n* = 5.

Q2. I have satisfactory opportunities for CPD, *n* = 437*, Prefer not to say *n* = 5.

Q3. My employer pays all costs required for me to engage in CPD, *n* = 436*, Prefer not to say *n* = 6.

Q4. My employer pays some of the costs required for me to engage in CPD, *n* = 437*, Prefer not to say *n* = 5.

Q5. I can use my work days to attend an event CPD, *n* = 437*, Prefer not to say *n* = 5.

Q6. I have access to online resources and courses, *n* = 437*, Prefer not to say *n* = 5.

Q7. I have no access to continued professional development *n* = 437*, Prefer not to say *n* = 5.

Total = the total number of people who answered Yes or No for the question. % = the amount of people who answered Yes (or No) for the question relative to the total number of people who answered Yes (or No) for the question.

**Figure 3:**
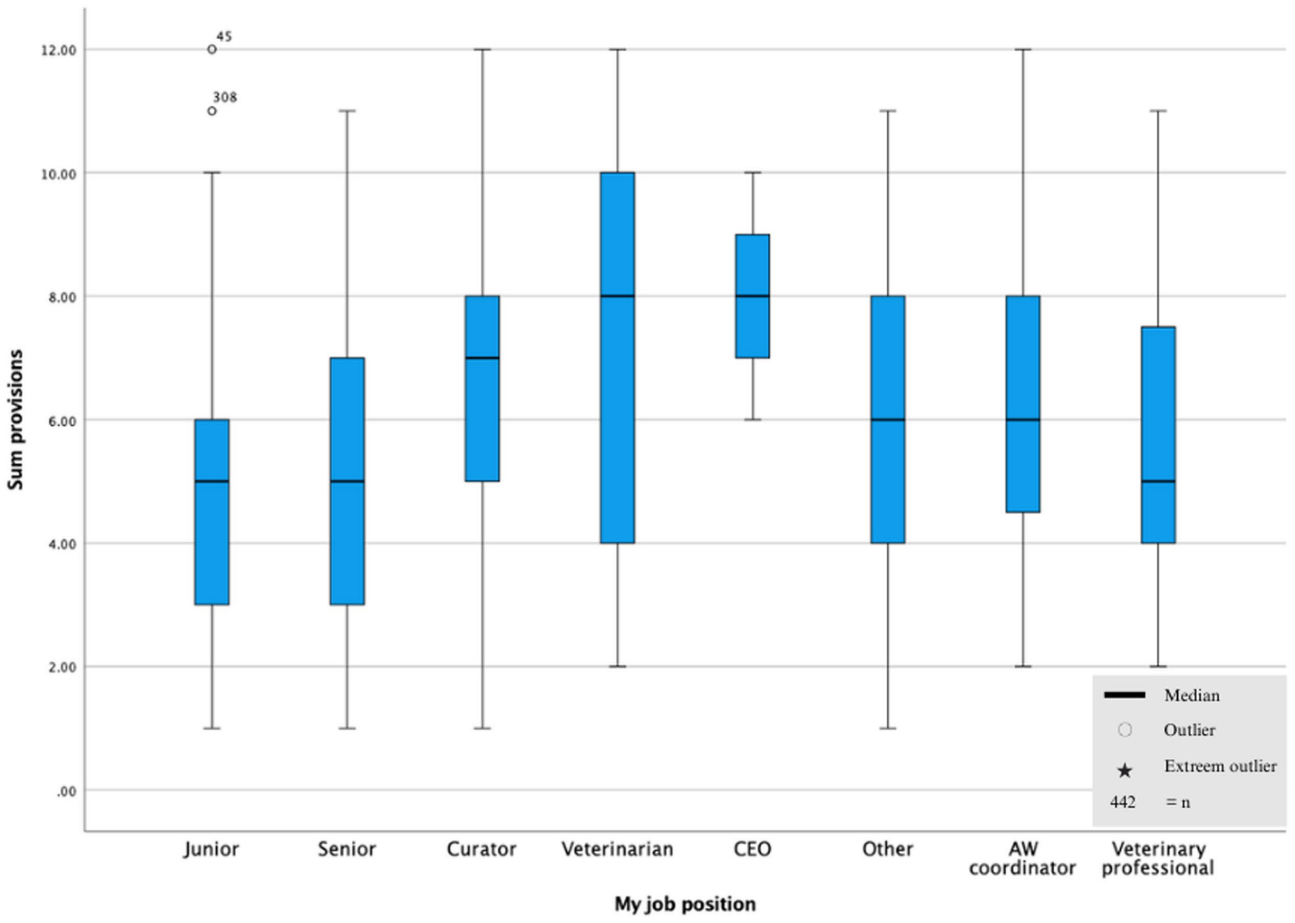
Variability of provisions for different job positions. The boxplot illustrates the variability of provisions for different job roles, with the high variations observed across all roles except the CEO. This boxplot includes outliers and extreme outliers due to the non-normal distribution of the data.

At Team level, participants reported receiving a mean of 5.5 *provisions* (SD = 2.56, 12). The boxplot in Figure 3 and Table 5 illustrates the variability of responses for sum of *provisions* across job positions.

**Table 5:** All provisions an organisation offers to employees per job position.

Question number	Response	Junior (*n* = 87)	Senior (*n* = 185)	Curator (*n* = 48)	Veterinarian (*n* = 15)	Veterinary professional (*n* = 19)	AW coordinator (*n* = 23)	CEO (*n* = 10)	Other (*n* = 50)	Total (*n* = *)
Q1	No	16 (18.6%)	33 (38.4%)	8 (9.3%)	2 (2.3%)	3 (3.5%)	9 (10.5%)	3 (3.5%)	12 (14%)	68 (100%)
	Yes	71 (20.2%)	152 (3.3%)	40 (11.4%)	13 (3.7%)	16 (4.6%)	14 (4%)	7 (2%)	38 (10.8%)	351 (100%)
Q2	No	33 (29.5%)	55 (49.1%)	5 (4.5%)	0 (0%)	5 (4.5%)	3 (2.7%)	1 (0.9%)	10 (8.9%)	112 (100%)
	Yes	54 (16.6%)	130 (40%)	43 (13.2%)	15 (4.6%)	14 (4.3%)	20 (6.2%)	9 (2.8%)	40 (12.3%)	325 (100%)
Q3	No	65 (18.8%)	144 (41.7%)	41 (11.9%)	10 (2.9%)	14 (4.1%)	19 (5.5%)	0 (0%)	42 (12.2%)	345 (100%)
	Yes	22 (24.2%)	41 (45.1%)	7 (7.7%)	5 (5.5%)	5 (5.5%)	4 (4.4%)	10 (2.9%)	7 (7.7%)	91 (100%)
Q4	No	55 (21%)	127 (48.5%)	27 (10.3%)	3 (1.1%)	9 (3.4%)	9 (3.4%)	1 (0.4%)	31 (11.8%)	262 (100%)
	Yes	32 (18.3%)	58 (33.1%)	21 (12%)	12 (6.9%)	10 (5.7%)	14 (8%)	9 (5.1%)	19 (10.9%)	175 (100%)
Q5	No	59 (20.6%)	138 (48.3%)	27 (9.4%)	6 (2.1%)	11 (3.8%)	13 (4.5%)	8 (2.8%)	24 (8.4%)	286 (100%)
	Yes	28 (18.5%)	47 (31.1%)	21 (13.9%)	9 (6%)	8 (5.3%)	10 (6.6%)	2 (1.3%)	26 (17.2%)	151 (100%)
Q6	No	54 (22.5%)	121 (50.8%)	18 (7.5%)	6 (2.5%)	11 (4.6%)	11 (4.6%)	1 (0.4%)	17 (7.1%)	240 (100%)
	Yes	33 (16.8%)	63 (32%)	30 (15.2%)	9 (4.6%)	8 (4.1%)	12 (6.1%)	9 (4.6%)	33 (16.8%)	197 (100%)
Q7	No	54 (22.5%)	113 (45.9%)	22 (8.9%)	7 (2.8%)	8 (3.3%)	15 (6.1%)	2 (0.8%)	25 (10.2%)	246 (100%)
	Yes	33 (16.8%)	72 (37.7%)	26 (13.6%)	8 (4.2%)	11 (5.8%)	8 (4.2%)	8 (4.2%)	25 (13.1%)	191 (100%)
Q8	No	33 (26.2%)	51 (40.5%)	9 (7.1%)	5 (4.0%)	6 (4.8%)	3 (2.4%)	4 (3.2%)	15 (11.9%)	126 (100%)
	Yes	54 (17.4%)	134 (43.1%)	39 (12.5%)	10 (3.2%)	13 (4.2%)	20 (6.4%)	6 (1.9%)	35 (11.3%)	311 (100%)
Q9	No	64 (20.6%)	141 (45.5%)	32 (10.3%)	9 (2.9%)	15 (4.8%)	12 (3.9%)	5 (1.6%)	32 (10.3%)	310 (100%)
	Yes	23 (18.1%)	44 (34.6%)	16 (12.6%)	6 (4.7%)	4 (3.1%)	11 (8.7%)	5 (3.9%)	18 (14.2%)	127 (100%)
Q10	No	77 (22%)	160 (45.7%)	35 (10%)	12 (3.4%)	15 (4.3%)	18 (5.1%)	3 (0.9%)	30 (8.6%)	350 (100%)
	Yes	10 (11.5%)	25 (28.7%)	13 (14.9%)	3 (3.4%)	4 (94.6%)	5 (5.7%)	7 (8%)	20 (23%)	87 (100%)
Q11	No	59 (28.5%)	86 (41.5%)	16 (7.7%)	9 (4.3%)	9 (4.3%)	10 (4.8%)	2 (1%)	16 (7.7%)	207 (100%)
	Yes	28 (12.2%)	99 (43%)	32 (13.9%)	6 (2.6%)	10 (4.3%)	13 (5.7%)	8 (3.5%)	34 (14.8%)	230 (100%)
Q12	No	57 (23.5%)	116 (47.7%)	18 (7.4%)	5 (2.1%)	14 (5.8%)	8 (3.3%)	1 (0.4%)	24 (9.9%)	243 (100%)
	Yes	30 (15.5%)	69 (35.9%)	30 (15.5%)	10 (5.2%)	5 (2.6%)	15 (7.7%)	9 (4.6%)	26 (13.4%)	194 (100%)

Q1. Appropriate clothing, *n* = 442.

Q2. Adequate equipment, *n* = 442.

Q3. Lunch, n = 441, Prefer not to say *n* = 1.

Q4. Tea and coffee n = 442.

Q5. Opportunities to participate in volunteering programs of choice, *n* = 442.

Q6. Team bonding exercises or events, *n* = 442.

Q7. Opportunities for awards of recognition for exceptional work, *n* = 442.

Q8. Access to health care, *n* = 442.

Q9. Self-care training, *n* = 442.

Q10. Access to a coach or trainer (e.g., when a skill needs to be mastered), *n* = 442.

Q11. Access to a counsellor (e.g., when a beloved animal passes away), *n* = 442.

Q12. A whistle-blower policy, *n* = 442.

Total = the total number of people who answered Yes or No for the question.

% = the amount of people who answered Yes (or No) for the question relative to the total number of people who answered Yes (or No) for the question.

The distribution of Provisions was significantly different from the normal distribution (W = 0.970, p < 0.001), skewed to the left, i.e., those reporting 5 provisions or less.

**Figure 4:**
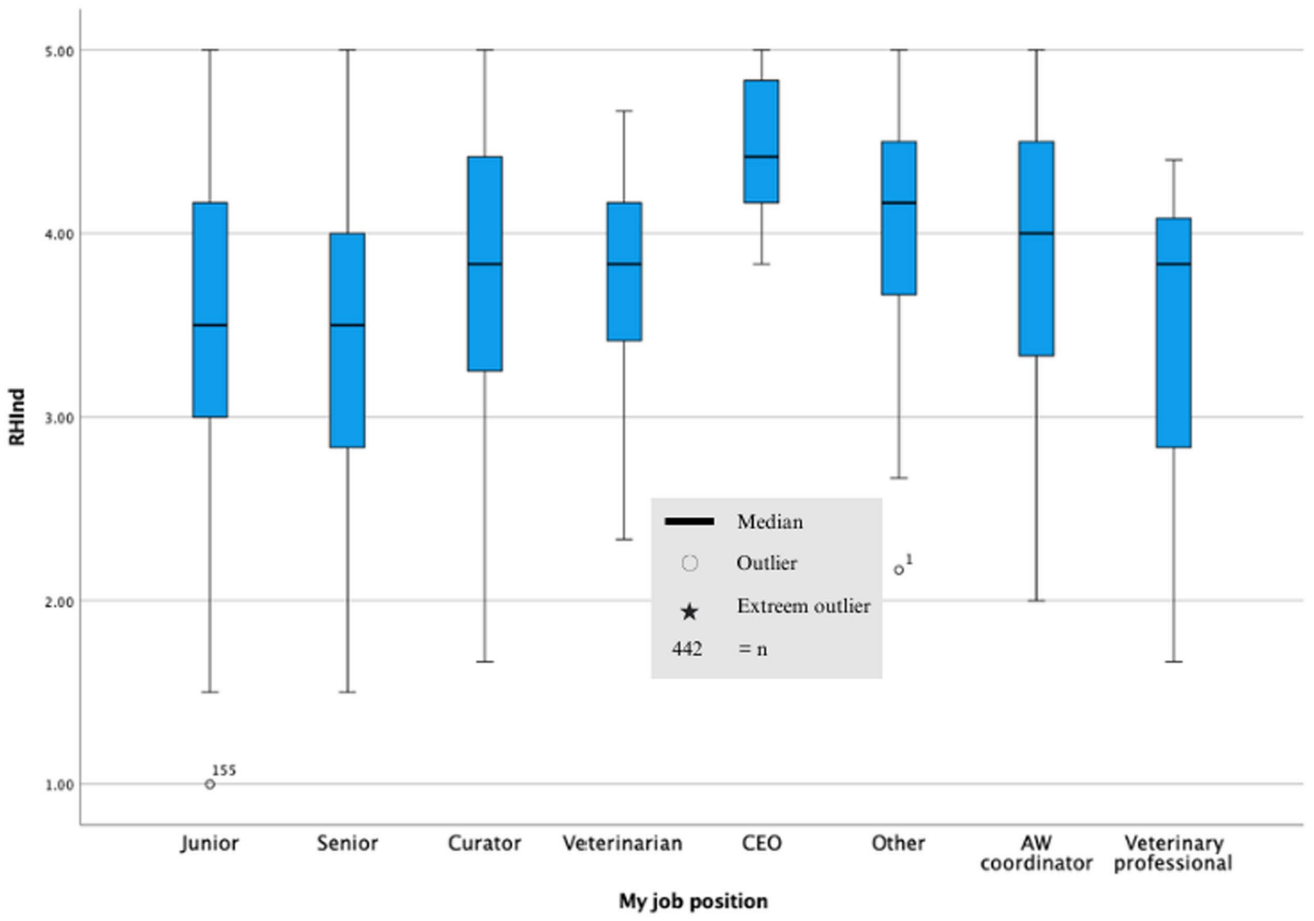
Variability mean of the questions RHInd per job position. The boxplot illustrates the variability of the mean of the questions related to the RHInd for different job roles, with the highest variations for juniors and seniors. This boxplot includes outliers and extreme outliers due to the non-normal distribution of the data.

For the six *RHInd* questions at Team level (n = 432), the boxplot in Figure 4 shows mean variability across job roles, with highest variations for *Juniors* and *Seniors*.

For Management-level, in relation to *Opportunities for development*, (which were different than the ones assessed on a team level) most participants (see all results Table 6, n = 112) responded that they get access to fully paid conferences/events (*n* = 93). Table 6 presents all results for management-level opportunities.

**Table 6:** All opportunities for manager.

Question number	Response	Senior (*n* = 185)	Curator (*n* = 48)	Veterinarian (*n* = 15)	Veterinary professional (*n* = 19)	AW coordinator (*n* = 23)	CEO (*n* = 10)	Other (*n* = 50)	Total (*n* = *)
Q1	No	14 (36.8%)	9 (23.7%)	2 (5.3%)	2 (5.3%)	2 (5.3%)	1 (2.6%)	8 (21.1%)	38 (100%)
	Yes	8 (10.8%)	28 (37.8%)	12 (16.2%)	1 (1.4%)	4 (5.4%)	8 (10.8%)	13 (17.6%)	74 (100%)
Q2	No	9 (34.6%)	6 (23.1%)	0 (0%)	3 (11.5%)	3 (11.5%)	0 (0%)	5 (19.2%)	26 (100%)
	Yes	13 (15.1%)	31 (36%)	14 (16.3%)	0 (0%)	3 (3.5%)	9 (10.5%)	16 (18.6%)	86 (100%)
Q3	No	18 (39.1%)	15 (32.6%)	3 (6.5%)	2 (4.3%)	2 (4.3%)	1 (2.2%)	4 (10.9%)	46 (100%)
	Yes	4 (6.1%)	22 (33.3%)	11 (16.7%)	1 (1.5%)	4 (6.1%)	8 (12.1%)	16 (24.2%)	66 (100%)
Q4	No	8 (42.1%)	7 (36.8%)	2 (10.5%)	1 (5.3%)	17 (6.6%)	0 (0%)	1 (5.3%)	19 (100%)
	Yes	14 (15.1%)	30 (32.3%)	12 (12.9%)	2 (2.2%)	6 (3.4%)	9 (9.7%)	20 (21.5%)	93 (100%)
Q5	No	15 (27.8%)	20 (37%)	9 (16.7%)	2 (3.7%)	2 (3.7%)	2 (3.7%)	4 (7.4%)	54 (100%)
	Yes	7 (12.1%)	17 (29.3%)	5 (8.6%)	1 (1.7%)	4 (6.9%)	7 (12.1%)	17 (29.3%)	58 (100%)
Q6	No	15 (27.8%)	20 (37%)	9 (16.7%)	2 (3.7%)	2 (3.7%)	2 (3.7%)	4 (7.4%)	54 (100%)
	Yes	7 (12.1%)	17 (29.3%)	5 (8.6%)	1 (1.7%)	4 (6.9%)	7 (12.1%)	17 (29.3%)	58 (100%)
Q7	No	16 (24.2%)	24 (36.4%)	9 (13.6%)	2 (3%)	3 (4.5%)	3 (4.5%)	9 (13.6%)	66 (100%)
	Yes	6 (13%)	13 (28.3%)	5 (10.9%)	1 (2.2%)	3 (6.5%)	6 (13%)	12 (26.1%)	46 (100%)
Q8	No	14 (26.4%)	19 (35.8%)	9 (17%)	1 (1.9%)	2 (3.8%)	1 (1.9%)	7 (13.2%)	53 (100%)
	Yes	8 (13.6%)	18 (30.5%)	5 (8.5%)	2 (3.4%)	4 (6.8%)	8 (13.6%)	14 (23.7%)	59 (100%)

Q1. Opportunities for continued personal and professional development, *n* = 112*, Prefer not to say *n* = 1.

Q2. Days off to attend event, *n* = 112*, Prefer not to say *n* = 1.

Q3. Fully paid conferences/events and days off to attend, *n* = 112*, Prefer not to say *n* = 1.

Q4. Fully paid conferences, *n* = 112*, Prefer not to say *n* = 1.

Q5. First aid, *n* = 112*, Prefer not to say *n* = 1.

Q6. People management skills, *n* = 112*, Prefer not to say *n* = 1.

Q7. Communication skills, *n* = 112*, Prefer not to say *n* = 1.

Q8. Human wellbeing courses such as occupational health and safety management, psychological health and safety, *n* = 112*, Prefer not to say *n* = 1.

Total = the total number of people who answered Yes or No for the question. % = the amount of people who answered Yes (or No) for the question relative to the total number of people who answered Yes (or No) for the question.

While not directly related to this hypothesis, to support an integrated approach to provisions that contribute to employee wellbeing, including finances, we report below on relevant findings related to finances and employees’ perspective on management understanding of employee’s needs.

Financial stability varied significantly by role (*n* = 437) (χ^2^ = 99.700, df = 21, *p* < 0.001). Senior and Junior caregivers together accounted for 81.6% of those reporting that their income was not nearly enough to cover basic expenses (*n* = 38), and represented the largest proportion of those only mostly making ends meet (*n* = 154). CEOs reported no financial difficulty, with all falling in the two highest categories (full results by role in Table 7).

**Table 7:** Finances to make it through the month per job position.

	Never (i.e., my income is not nearly enough to cover my basic expenses) (*n* = 38)		Mostly (i.e., my income covers most of my basic expenses, but sometimes it is a bit short) (*n* = 154)		Completely (i.e., my income always covers my basic expenses but there is little left for anything else) (*n* = 135)		Comfortably (i.e., all basic expenses are covered and some of my income can be spend on wants rather than needs) (*n* = 110)	
CEO	0	0%	0	0%	1	1.2%	9	8.2%
Other	4	10.5%	11	7.1%	8	9.4%	23	20.9%
Veterinarian	1	2.6%	1	0.6%	6	4.4%	7	6.4%
Veterinary professional	2	5.3%	10	6.5%	5	3.7%	2	1.8%
AW coordinator	0	0%	5	3.2%	8	5.9%	10	9.1%
Curator	0	0%	8	5.2%	17	12.6%	23	20.9%
Senior caregiver	16	42.1%	81	52.6%	59	41.2%	29	26.4%
Junior caregiver	15	39.5%	38	24.7%	27	20%	27	31.8%
Prefer not to say	5	1.1%	5	1.1%	5	1.1%	7	6.4%

Total sample size is 437. Pearson Chi-Square = 99.700, df = 21, *p* = <0.001.

**Figure 5:**
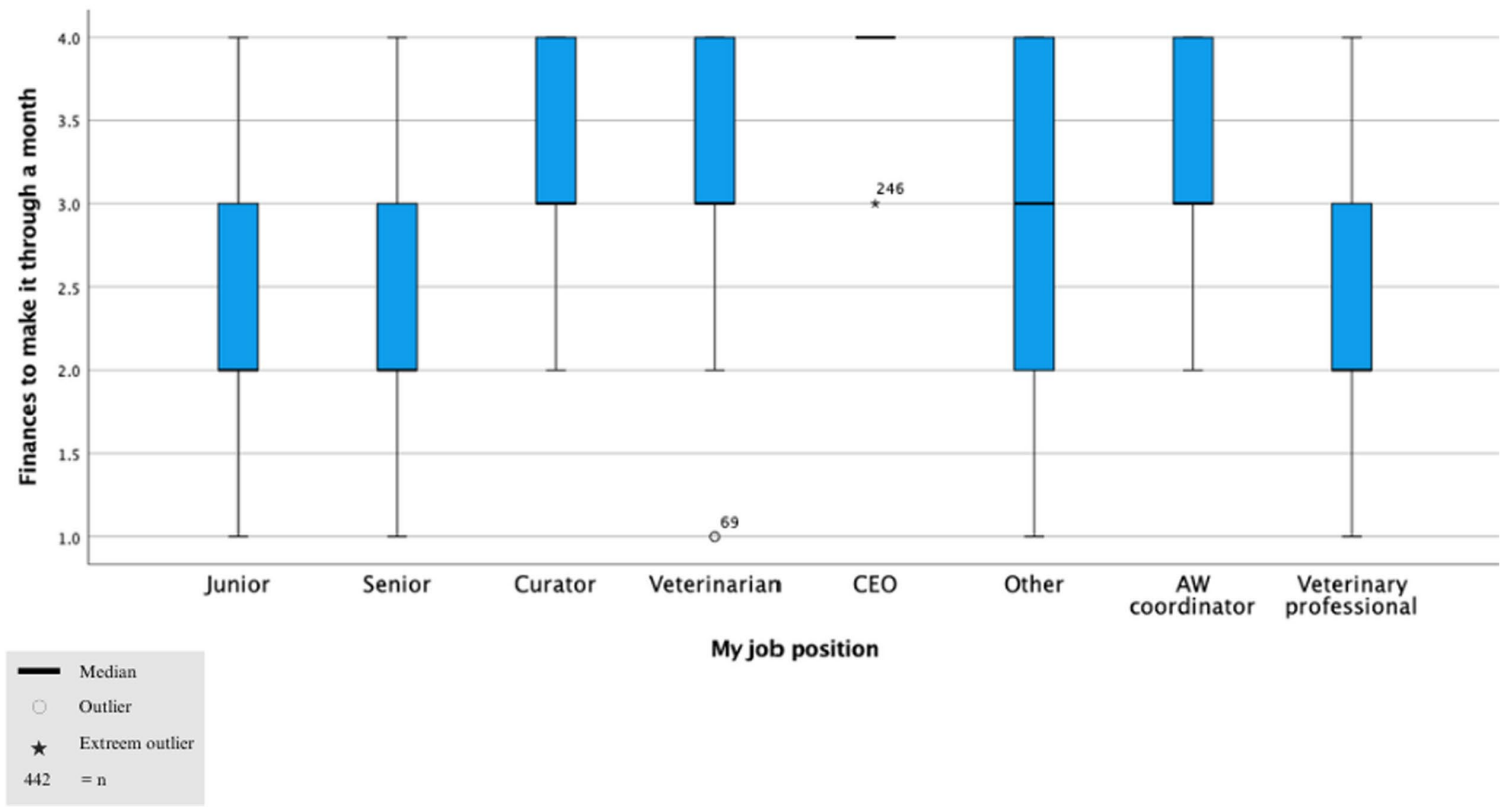
Variability of finances per job position. Figure 3 illustrates the variability of one’s ability to make it through the month financially for different job positions, with the highest variations for juniors, seniors, veterinary professionals, and other. This boxplot includes outliers and extreme outliers due to the non-normal distribution of the data 4 is the highest which explains the lack of whiskers/partly lack of whiskers for some job positions.

Figure 5‘s boxplot shows variability in financial stability across job roles, with highest variations among *Juniors, Seniors, Others,* and *Veterinary professionals*.

Opinions (n = 437) on financial compensation were closely divided but skewed toward disagreement (χ^2^ = 135.901, df = 28, *p* < 0.001): 49.9% expressed some disagreement (strongly disagree: *n* = 99, 22.7%; somewhat disagree: *n* = 119, 27.3%), 6.9% neither agreed nor disagreed (*n* = 30), and 41.4% expressed some agreement (somewhat agree: *n* = 130, 29.7%; strongly agree: *n* = 51, 11.7%). Role differentials were stark: Senior and Junior caregivers together accounted for 79.8% of those strongly disagreeing, while no CEO fell in any disagree category (full breakdown in Table 8).

**Table 8:** Adequate financial compensation per job position.

	Strongly disagree		Somewhat disagree		Neither agree nor disagree		Somewhat agree		Strongly agree	
CEO	0	0%	0	0%	0	0%	3	2.3%	7	13.7%
Other	4	4%	7	5.9%	2	6.7%	20	15.4%	17	33.3%
Veterinarian	5	5.1%	5	4.2%	0	0%	1	0.8%	4	7.8%
Veterinary professional	4	4%	9	7.6%	1	3.3%	3	2.3%	0	0%
AW coordinator	3	3%	1	0.8%	2	6.7%	13	10%	3	5.9%
Curator	4	4%	9	7.6%	4	4%	22	16.9%	7	13.7%
Senior caregiver	44	44.4%	58	48.7%	16	53.3%	56	43.1%	10	19.6%
Junior caregiver	35	35.4%	30	25.2%	5	16.7%	12	9.2%	3	5.9%
Prefer not to say	8	1.8%	8	1.8%	8	1.8%	8	1.8%	8	1.8%
Total	99	100%	119	100%	30	100%	130	100%	51	100%

*N* = 437. Pearson Chi-Square = 135.901, df = 28, *p* = <0.001. W = 0.834, *p* < 0.001 and skewed toward those who somewhat agreed to receive adequate compensation.

Most participants worked some form of unpaid overtime: 40.9% often worked additional unpaid hours (*n* = 178) and a further 39.5% sometimes did so (*n* = 172), with only 19.5% (*n* = 85) reporting they worked only paid hours (χ^2^ = 49.434, df = 14, *p* < 0.001). Unpaid overtime was concentrated among Senior and Junior caregivers, who together represented 55.3% of those often working unpaid and 72.7% of those sometimes doing so (full results by role in Table 9).

**Table 9:** When at work working hours per job position.

	I often work additional hours I am not paid for (*n* = 178, 40.9%)		I sometimes work additional hours I am not paid for (*n* = 172, 39.5%)		I only work the hours I am paid for (*n* = 85, 19.5%)	
CEO	9	5.1%	0	0%	1	1.2%
Other	49	11.3%	15	8.7%	8	9.4%
Veterinarian	7	3.9%	8	4.7%	0	0%
Veterinary professional	7	3.9%	7	4.1%	3	6.4%
Animal welfare scientist/coordinator	11	6.2%	7	4.1%	5	5.9%
Curator	33	18.5%	10	5.8%	5	5.9%
Senior animal caregiver	62	34.8%	88	51.2%	35	41.2%
Junior animal caregiver	23	1.9%	37	21.5%	27	31.8%
Prefer not to say	3	0.7%	3	0.7%	3	0.7%

*N* = 435. Pearson Chi-Square = 49.434, df = 14, *p* = <0.001.

For the question *When at work* (*n* = 435, see Table 9), participants most often worked additional unpaid hours (*n* = 178, 40.9%).

To the question, *the organisation has a good understanding of the needs and expectations of the staff*, most managers (*n* = 113) answered yes (*n* = 98, 86.7%), followed by I do not know (*n* = 13, 11.5%), no (*n* = 1, 0.9%), and one person (0.9%) selected the *prefer not to say* option.

### 3.3 Hypothesis 2

Hypothesis 2 predicted that levels of self-efficacy and experience mediates/moderates* the relationship between good working conditions (provisions/opportunities) and *Indjobsat*, *Orgjobsat*, and *life satisfaction*. We found partial support, more for *self-efficacy* than for *experience*.

*Mediation Self-efficacy*: Analyses showed *self-efficacy* was a significant mediator between sum of *provisions* and mean *Orgjobsat* (0.007 (effect value); CI: 0.0017–0.156) and between sum of *provisions* and *life satisfaction* (0.06; CI: 0.01–0.12), both at 95% confidence level. For coefficients, see Figures 1a,b. *Self-efficacy* was not a significant mediator between *provisions* and *Indjobsat* (0.006; CI: −0.0004–0.14).

*Experience*: See coefficients of direct relationships tested for mediation of *provisions* and *life satisfaction* in Figure 1c. The indirect effect of *experience* was significant, with effect value 0.027 (CI: 0.001–0.63) at 95% confidence level. The other two mediation analyses were not significant.

*Moderation*: For the tested moderation models, only *self-efficacy*’s moderation between *provisions* and *Orgjobsat* was significant. There was no direct relationship between *self-efficacy* and *Indjobsat*, or between *provisions* and *life satisfaction*. The positive interaction effect suggests that a higher sum of provisions leads to higher *Orgjobsat*, especially with high levels of self-efficacy. At all levels of *self-efficacy* (coefficient for low self-efficacy = 0.18, coefficient for medium self-efficacy = 0.22; coefficient for high self-efficacy = 0.27, 46.00), a significant relationship existed between *provisions* and *Orgjobsat*, becoming stronger with increasing levels of *self-efficacy*. None of the other moderations were significant.

### 3.4 Hypothesis 3

Hypothesis 3 predicted that individuals reporting good individual and team engagement would report higher Indjobsat, Orgjobsat, and life satisfaction.

At both Team and Managerial levels, support for H3 was found, with significant, positive correlations between mean *individual* and *team* engagement and all 4 outcome variables (see Table 10 for Team level (*n* = 426–442) and Table 11 for Managers level (*n* = 113)). Across both levels, employees somewhat agreed to perceiving good *individual* and *team* engagement. Spearman’s correlation analysis showed a strong positive significant relationship *individual* and *team* engagement with levels of *Indjobsat* and *Orgjobsat*. A medium positive relationship between *team* engagement and life satisfaction levels and a strong positive relationship between *individual* engagement and *life satisfaction* were found. A weak positive significant relationship between *individual* and *team* engagement and *self-efficacy* was found. The descriptive statistics and distribution of the key variables for H3 can be found in Table 12.

**Table 10:** Spearman rho correlations life satisfaction, *Indjobsat*, *Orgjobsat*, individual and team engagement, and self-efficacy.

Variables	N	1	2	3	4	5	6
1 Mean organisational job satisfaction	440	1.00	0.62**	0.44**	0.77**	0.66**	0.19**
2 Individual job satisfaction	442		1.00	0.47**	0.59**	0.55**	0.16**
3 Total life satisfaction	426			1.00	0.44**	0.36**	0.30**
4 Mean individual engagement	442				1.00	0.69**	0.24**
5 Mean team engagement	442					1.00	0.17**
6 Total self-efficacy	426						1.00

Correlation is significant at the 0.01 level (2-tailed) **p < 0.01. Samples sizes are based on the amount of people who fully completed the relevant questions.

**Table 11:** Spearman rho correlations managers only for life satisfaction, *Indjobsat*, *Orgjobsat*, individual and team engagement, self-efficacy, sum of processes.

Variables	N	1	2	3	4	5	6	7
1 Mean organisational job satisfaction	112	1.00	0.66**	0.51**	0.77**	0.63**	0.37**	0.30**
2 Individual job satisfaction	113		1.00	0.50**	0.62**	0.55**	0.35**	0.38**
3 Total life satisfaction	113			1.00	0.49**	0.41**	0.45**	0.12
4 Mean individual engagement	113				1.00	0.65**	0.34**	0.27**
5 Mean team engagement	113					1.00	0.38**	0.20*
6 Total self-efficacy	113						1.00	0.16
7 Sum of processes	113							1.00

Correlation is significant at the 0.01 level (2-tailed) *p < 0.05, **p < 0.01. Samples sizes are based on the amount of people who fully completed the relevant questions.

**Table 12:** Understanding individual and team engagement, manager observations of team(s), and *RHInd*.

Series of questions reflecting 1 underlying concept	Cronbach alpha	Mean	Median	SD	Skewed	N	*p*-value
Individual engagement	0.79	3.79	3.89	0.71	To the right, i.e., those reporting good individual engagement	W = 0.961	*p* < 0.001
Team engagement	0.88	3.84	3.90	1.07	To the right, those reporting good team engagement	W = 0.959	*p* < 0.001
*RHInd*	0.80	3.58	3.66	0.83	To the right, i.e., those reporting good organisational approaches	W = 0.972	*p* < 0.001
Diverse set of 15 questions related to manager observations	0.93	35.84	35.00	12.83	To the right, somewhat agree	W = 0.97	*p* = 0.19

The *p*-value reflects the Shapiro–Wilk test of normality (W) for each variable. Theoretical score ranges: individual engagement (mean of 5 items, possible range 1–5); team engagement (mean of 5 items, possible range 1–5); RHInd (mean of 6 items, possible range 1–5); manager observations (sum of 15 items, 1–5 per item, possible range 15–75). Sample sizes: individual engagement, team engagement, and RHInd n = 432–442 (all participants); manager observations *n* = 113 (managers only).

### 3.5 Hypothesis 4

Hypothesis 4 predicted that levels of self-efficacy and experience mediates/moderates* the relationship between team engagement and *Indjobsat*, *Orgjobsat*, and *life satisfaction*.

**Figure 6:**
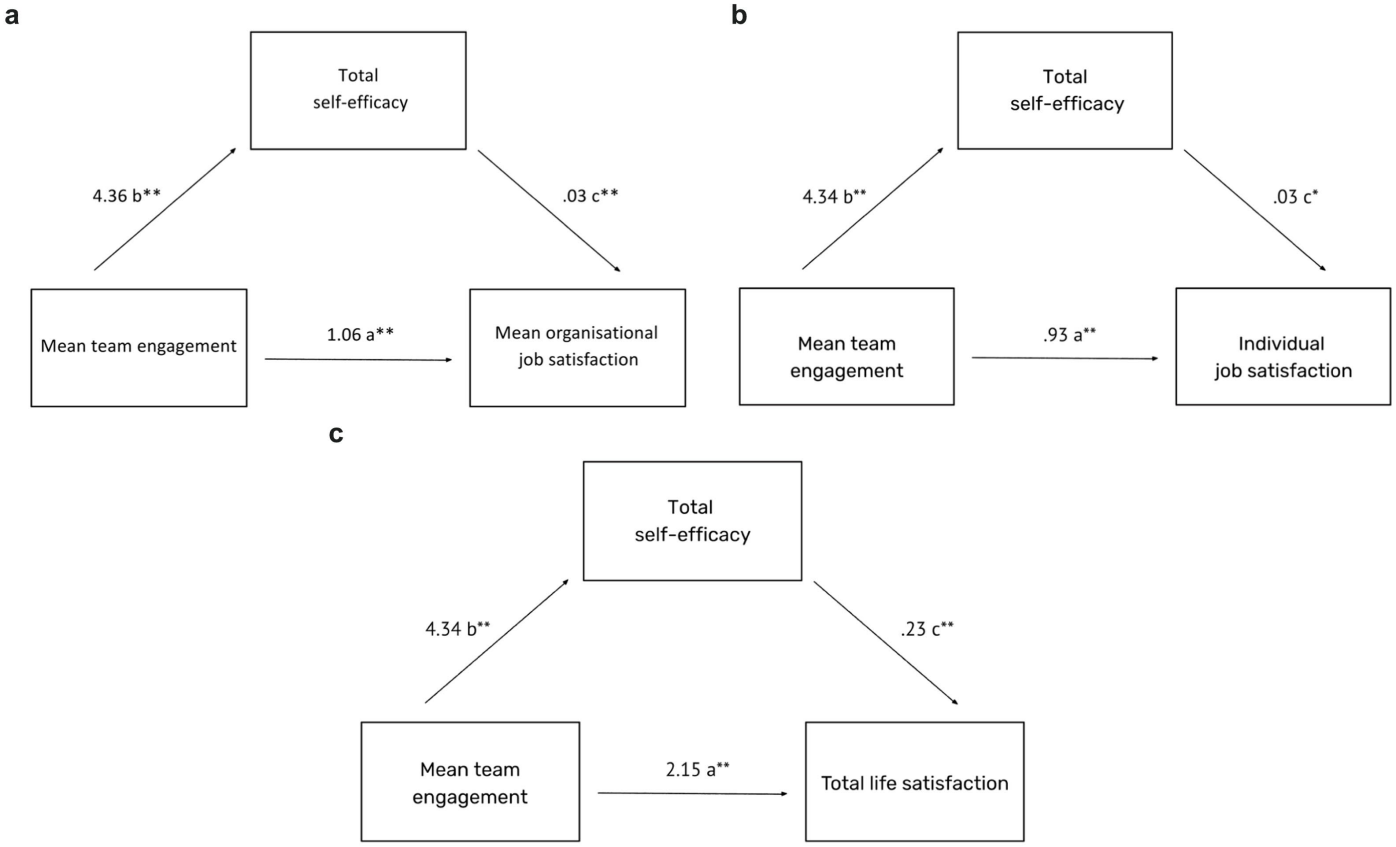
**(a)** Self-efficacy, mean team engagement, and mean organisational job satisfaction. This figure illustrates the mediation model for the mean team engagement, mean organisational job satisfaction, and total self-efficacy. * = *p* < 0.05 ** = *p* < 0.001. **(b)** Self-efficacy, mean team engagement, and mean individual job satisfaction. This figure illustrates the mediation model for the mean team engagement, individual job satisfaction, and total self-efficacy. * = *p* < 0.05 ** = *p* < 0.001. **(c)** Self-efficacy, Mean Team Engagement, and Mean Individual Job Satisfaction This figure illustrates the mediation model for the mean team engagement, total life satisfaction, and total self-efficacy. * = *p* <0.05 ** = *p* <0.001.

Support for this hypothesis was only found for s*elf-efficacy.* Mediation analyses identified that *self-efficacy* was a significant mediator in the relationship between *team* engagement and *Orgjobsat* (0.11; CI: 0.01–0.23), as well as between *team* engagement and *life satisfaction* (1.0076; CI: 0.42–1.73), and *self-efficacy* was a significant mediator in the relationship between *team* engagement and *Indjobsat* (0.12; CI: 0.001–0.3010), all at 95% confidence level. See Figures 6a–c for all coefficients. The mediations for *experience* were not significant, and none of the moderations were significant.

### 3.6 Hypothesis 5

This section includes manager questions related to *RHOrg*, perceptions regarding working with their teams and management, ISO certifications, and charters.

Hypothesis 5 predicted that managers reporting more holistic employee wellbeing processes (*holprocesses*) would report higher Indjobsat, Orgjobsat, and life satisfaction.

At the Managerial levels, H5 was mostly supported with exception of the relationship with life satisfaction. Table 13 (*n* = 113) illustrates a Spearman’s correlation showing a weak positive significant relationship sum of *holprocesses* with levels of *Indjobsat and Orgjobsat*. A weak positive significant relationship sum of *RHOrg* with levels of *Indjobsat, and life satisfaction* was also found.

**Table 13:** Spearman rho correlations managers only for life satisfaction, *Indjobsat, Orgjobsat*, mean *RHOrg*, and sum of *Holprocesses*.

Variables	N	1	2	3	4	5
1 Mean organisational job satisfaction	112	1.00	0.66**	0.51**	0.20*	0.30**
2 Individual job satisfaction	113		1.00	0.50**	0.24*	0.28**
3 Total life satisfaction	113			1.00	0.21*	0.12
4 RHOrg	113				1.00	0.22*
5 Sum of holprocesses	113					1.00

Correlation is significant at the 0.01 level (2-tailed) *p < 0.05, ** *p* < 0.01.

A PCA (see Table 14 for all factor loadings, *n* = 109) was conducted for questions related to the *RHOrg,* which included questions regarding organisational philosophy and ways of working. The Cronbach’s alpha for these questions was 0.66. The mean was 3.24 (SD = 0.45) and the median was 3.13. Three components were found, and upon discussion with a Rhinelands practitioner, three terms matched the item loadings across the components: (1) Leadership (organisation), (2) Management control (outcomes), and Self-efficacy. Leadership included aspects such as leadership style (top-down, hierarchy) and understanding of expertise on the work floor. Management control included being results-oriented and individual performance. Self-efficacy included self-management and trust. This preliminary exploration of the *RHOrg* approach requires further research but represents an important first step.

**Table 14:** *RHOrg* principal component analysis.

Leadership (organising)	Management control (outcomes)	Self-efficacy
Craftsmanship is at the heart of our business (0.685)	We work with corporate targets and staff will be judged on them (0.678)	Professionals and teams of professionals are self-managing (0.666)
Hierarchy does not mean much to us (0.525)	We work in a very results-oriented way (0.668)	We are based on trust, an agreement is an agreement. Planning, control, and management systems are therefore secondary (0.664)
The dominant leadership style is top-down (−0.552)	Who’s in charge gets the final say, instead of who knows (*the answer*), gets to the final say (0.591)	
Measurable objectives play an important role for us (0.599)	Customers are ultimately more important than employees (−0.561)	
Sustainability and corporate social responsibility are serious strategic issues for us (0.651)	The individual performance, that’s what matters (0.621)	
Our top managers steer more towards the short term than the long term (−0.595)		
There is a lot of consultation because we think consensus is important (0.798)		
Managers understand the expertise on the work floor (0.767)		
Ultimately, the organisation is there for the people in the organisation, (and the people are not there for the organisation) (0.497)		

Cronbach alpha for this set of questions was 0.663. Four participants left answers blank.

Questions related to the managers’ perceptions of working with their teams and their direct management are highlighted in Table 15 (*n* = 113). Key findings include employees feeling strongly supported by both their team and higher management and only feeling somewhat trapped.

**Table 15:** Manager perceptions working with their team(s) and management.

Idem	SD	SWD	NAND	SA	STA	IDK	N	*P*-value	Skew
Perceptions									
I feel supported by the team(s) I supervise, e.g., good communication, getting the job done together	0 (0%)	7 (6.2%)	5 (4.4%)	43 (38.1%)	58 (51.3%)	0 (0%)	W = 0.723	*p* < 0.001	To those feeling supported by the team.
I feel supported by upper management, e.g., clear goals, budget	0 (0%)	19 (16.8%)	9 (8%)	34 (30.1%)	42 (37.2%)	0 (0%)	W = 0.823	*p* < 0.001	To those feeling strongly supported by their management
I feel trapped between the team(s) I supervise and upper management (e.g., conflicting goals, poor communication)	19 (16.8%)	27 (23.9%)	18 (15.9%)	37 (32.7%)	10 (8.8)	2 (1.8%)	W = 0.888	*p* < 0.001	To those somewhat agreeing to feeling trapped between the team they supervise and upper management.
I feel supported by other managers in the organisation (e.g., for advice, exchange)	2 (1.8%)	3 (2.7%)	9 (8%)	44 (38.9%)	53 (46.9%)	2 (1.8%)	W = 0.745	*p* < 0.001	To those somewhat agreeing to feeling supported by other managers in the organisation.
I think I have a clear understanding of the needs of the team(s) I supervise (e.g., resources needed, animal needs)	0 (0%)	2 (1.8%)	4 (3.5%)	36 (31.9%)	70 (61.9%)	1	W = 0.667	*p* < 0.001	To those strongly agreeing to having a clear understanding of the needs of the team(s) I supervise (e.g., resources needed, animal needs).

Codes are: SD, Strongly disagree; SWD, Somewhat disagree; NAND, Neither agree nor disagree; SA, Somewhat agree; STA, Strongly agree; IDK, I do not know.

For questions with participants in a managerial role (*n* = 113) related *holprocesses* such as ISO45001 certifications, and existence of animal and human wellbeing charters at the organisation, see Table 16 and for ISO45003 (*n* = 112) see Table 17. With respect to ISO45003, promoting health and safety and reducing psychosocial hazards, most participants somewhat agreed that their organisation’s processes were satisfactory, with space for improvement. Regarding worker consultations about position and work including job satisfaction, work conditions and development, most participants (*n* = 113) in managerial roles answered yearly (*n* = 37, 32.7%), followed by other (*n* = 35, 31%), quarterly (*n* = 27, 23.9%), I do not know (*n* = 13, 11.5%), and 1 person (0.9%) selected Prefer not to say. Participants specified comments such as bi-yearly (*n* = 17) and less frequent responses including rarely, occasionally, every 2 y, monthly 1-on-1’s, and that survey results are often not distributed or followed up on. Five participants reported no consultations.

**Table 16:** ISO and charter related questions at managerial level.

Question	Yes	No	I do not know	In process of development	Prefer not to say	Total
Is the organisation ISO 45001:2018—occupational health and safety management systems certified?	24 (21.2%)	14 (12.4%)	74 (65%)	0 (0.0%)	1 (0.9%)	113
Is the organisation ISO 45003:2021—occupational health and safety management —psychological health and safety at work certified?	12 (10.6%)	17 (15%)	83 (73.5)	0 (0.0%)	1 (0.9%)	113
The organisation has a whistle-blower policy	72 (63.7%)	8 (7.1%)	31 (27.4%)	1 (0.9%)	1 (0.9%)	113
The organisation has a written human wellbeing charter; stipulating details of the philosophy, ethics, and practical conduct of promoting good human wellbeing	36 (31.9%)	28 (24.8%)	46 (40.7%)	0 (0.0%)	3 (2.7%)	113
The organisation has a written animal welfare charter; stipulating details of the philosophy, ethics, and practical conduct of promoting good animal welfare	98 (86.7%)	1 (0.9%)	13 (11.5%)	0 (0.0%)	1 (0.9%)	113

*N* = 113.

**Table 17:** ISO45003 questions at managerial level.

Idem	Cronbach alpha	Mean	Median	SD	Skewed
ISO45003 the risks and opportunities, including psychosocial hazards	0.89	3.90	4	0.88	To the right, somewhat agree
ISO45003 rehabilitation and return-to-work programmes	0.88	4.26	4.25	1.07	To the right, somewhat agree
ISO45003 health, safety and well-being at work	0.87	4.40	4.5	1.20	To the right, somewhat agree
ISO45003 internal audits	0.93	4.04	4	1.96	To the right, somewhat agree
ISO45003 appropriate communication	0.88	3.73	3.83	1.18	To the right, somewhat agree

*N* = 112. Combined multiple sub questions into a set of questions to calculate the Cronbach alpha, mean, median, and SD.

## 4 Discussion

This research examines how zoo professionals in ZOAQs perceive their *life satisfaction*, *Indjobsat* and *Orgjobsat*, and how *self-efficacy, experience, team engagement, processes, provisions, opportunities*, and ways of working aligned with Rhinelands (*RHInd/RHOrg*) contribute to these perceptions. Correlations, mediations, and moderations were used for finances, *opportunities, provisions, holprocesses*, and measures related to *RHInd* and PCA related to *RHOrg* were explored. The discussion is structured as an interconnected whole, with interview quotes highlighting the findings.

The correlation analysis revealed strong positive relationships between *provisions* and *opportunities* with *Orgjobsat* levels and between mean *RHInd* and *Orgjobsat* levels, likely because support individuals identify as enhancing their job performance and self-efficacy. This aligns with the strong positive relationship between mean *RHInd* and *Indjobsat* levels and the moderate positive relationship between *provisions* and *opportunities* and *Indjobsat* levels, suggesting work perceptions and organisational appreciation are valued above access to resources.

S*elf-efficacy* mediated the relationship between *provisions* and *Orgjobsat* and between *provisions* and *life satisfaction*, but not *Indjobsat*. *Experience* only mediated the relationship between *provisions* and *life satisfaction*. The moderation analysis showed only one significant relationship remained: between *self-efficacy* and *provisions* and *Orgjobsat*. The positive interaction effect indicates higher *provisions* correlate with higher *Orgjobsat*, especially at high levels of *self-efficacy*.

While most somewhat agreed to experiencing *Indjobsat*, the nature of the profession and challenges that come with it such as low financial reward, limited resources, demanding working environments (1) stresses the importance of creating a culture of care, including improving working conditions.

### 4.1 Working conditions

Less than half of the respondents felt that they had satisfactory *opportunities*, suggesting that on-the-job training, online resources, and CPD may not meet their needs to feel competent, confidents, and or informed. At the managerial level, only slightly more than half received training in people management and occupational safety, while less than half received communication skills training (see Table 2). Most managers received people management courses as they advanced, but one-third received no such training. About 7% of respondents lacked access to continued professional development across all roles except *Curators, Veterinarians,* and *CEO*. While this study did not directly measure competence levels or care quality outcomes, the relationship between inadequate training access and professional effectiveness in caring roles is well established in the wider literature (1, 85–87), particularly in a field that remains largely unregulated and unstandardised, where formal educational pathways frequently do not align with the knowledge and skills required in practice.

For *provisions* like appropriate clothing, coffee and tea, or team bonding, participants reported to only have on average 5 out of 12 relevant *provisions*. Healthcare, or employer offered healthcare, access was not universally available across the sample. Given variation in national healthcare systems across the seven participating countries, including contexts where employer-sponsored healthcare is not universally mandated, this likely reflects structural differences between national systems rather than organisational policy alone. Individual participants cannot be linked to specific countries in this anonymised dataset, and country-level explanations remain inferential. Further, access to a coach for mastering new skills was missing across roles, while access to a counsellor when an animal passed away was available to all. The knowledge of a whistleblower policy was lacking across roles, except at the managerial level. Differences in *provision*s existed between job roles, with volunteering programs, team bonding events, and recognition awards mainly available to managers. To support employees in ZOAQs, understanding and facilitating important provisions at all levels is essential to retain expertise, deliver goals, and support wellbeing, including attention to supportive processes.

Indeed, the correlation analysis shows weak positive significant relationships between the sum of *holprocesses* and levels of *Indjobsat* and *Orgjobsat (at managerial level),* suggesting that while somewhat satisfactory, they do not substantially contribute to either measure. This suggests that while processes exist, they may not align with employee values, goals, and needs (88), increase bureaucracy (89), or truly support the nature and dynamics of the work environment (90). Furthermore, many managers did not seem to be aware of *holprocesses* (e.g., ISO related), particularly those related to human wellbeing, that existed in their organisation, possibly explaining the weak relationship.

Organisational charters or other guiding documents should shape an organisation’s culture, ethical standards, operational approaches, identity, and alignment with core values (53, 91). Those facilitating collective goals should be aware of and implement, guide, enforce, and amend processes with input from affected parties to ensure that they are workable and meaningful (92). For example, incentives and provisions which may not support employees, as one interviewee stated, “The fee for transport was a lot of money out of my paycheck, and even with transport benefits it was too high, so I moved out of the city, which made me wonder, is that sort of benefit actually helpful? (108, X), and “They brought in flexible hours, but that’s only really relevant to those that work in the office or can work from home “(140, Senior).

Similarly, as we consider animals holistically, meaningful processes and opportunities are essential for supporting employee wellbeing and sustainable programs (1, 93). Consistent with the wider literature (9, 94), it is important to acknowledge how multiple effects on employees’ wellbeing, such as having multiple jobs, inflation, extended travel time due to expensive urban living costs, or inability to save money, can contribute to staff turnover and prevent people from reaching their full potential.

Promoting employee wellbeing through Diversity, Equity, Equality, and Inclusion (DEEI) is essential to creating a culture where people feel respected and understood (95), while supporting collective goals. Pandey and Ganatra (96) found that cultural stereotypes, personal biases, and institutionalised practices are the main sources of HR biases. DEEI training, bias awareness, standardised HR processes, and technology have been proposed to reduce biases and promote workplace equality (96, 97). Addressing discrepancies in provisions are essential to creating a more just workplace (48, 98, 99), and reducing pay gaps (100), such as leaders earning 15 times more than animal caregivers as some of the interviewees eludes to:

We also see how much absentee owners, our hiring staff, or our CEO makes. And it’s just vastly different than what we make. As salaries of those in top positions are public, I know our CEO makes 15 times more than what I earn (108, Senior).

Unfair opportunities and perceptions of unfairness can significantly affect employee morale, productivity, and organisational performance (101). Limited access to meaningful CPD and a lack of foundational processes do not provide a foundation for a sustainable organisation. Data-driven approaches with systematic information collection support evidence-based decision-making, enhance the identification of unfair opportunities in job positions (102), and mitigate bias (103). However, this remains beyond individual employee control, which explains why experience mediates the association between *provisions* and *life satisfaction* but not other factors. Through experience, employees can maximise available provisions affecting life satisfaction; however, if factors contributing to *Indjobsat* are uncontrollable, *experience* cannot compensate.

Participants indicated they somewhat disagreed with receiving adequate financial compensation, further supporting the weak relationship between *provisions* and *life satisfaction* levels. This suggests misalignment between employee needs and provisions like clothing, healthcare access, and unpaid overtime, an interviewee noted, “I’m leaving like 15 0r 20 minutes late every day” (56, Junior), affects their personal lives. While animal welfare work is not chosen for financial rewards (1, 2), excessive work load, financial strain causes stress, burnout, and poor wellbeing (104–106), leading to high turnover (94).

Organisational culture, job satisfaction, advancement opportunities, and value alignment can reduce the effects of low wages on turnover (94). Work-life balance and job security can intensify the impact of low wages on turnover. Roslinda and Andias (107) found that good wages are the most influential factor across all sectors. One participant stated, “It paid so little money that I had to locum at weekends doing emergency vet work almost every weekend, and I was completely exhausted (119, Veterinarian: has since left the zoo field). Another interviewee stated:

The people that are left here are more burned out because of low staff numbers and positions removed during COVID not being filled, while adding expectations and programs. They had a compensation review, and we did not get a raise and it still is not a liveable wage for X. I live two hours away and I do not get paid for that (154, Senior).

My commute is about an hour and 20 min, I take the train, I walk for about 20 min, and then I drive like to the station but I do not really count that, but I do not get paid for my travel time (27, Junior).

Employee turnover is expensive and affects the remaining staff; therefore, minimising it is crucial (108). Wei and Ayo (109) found that training initiatives need better alignment with organisational objectives, and organizational culture requires clearer task focus to enhance employee productivity and retention. It is noteworthy that seven of the 39 interview participants had left the ZOAQ field by the time of writing. While this was not a planned outcome measure, it is a meaningful observation that contextualises the challenges described throughout this study.

Supporting employee wellbeing and collective goals requires understanding both, including changes in a dynamic organisational landscape (110, 111). When asked if managers think the organisation understands staff needs and expectations, most agreed, though 13% indicated they did not know. Ten percent were unaware of consultations, while some noted ‘every few years’ or that survey results were not followed up on. Leaders know that facilitators need connection with frontline staff to understand challenges and recognise successes. Examples of diverse opinions from interviewees are:

I try and spend an hour with each team every few months, just talking to them, listening to their concerns, hearing their ideas. And I make sure I go for a walk every day and just see what the animals are doing, what the habitats are doing (314, CEO).

Management creates their own bubble, that’s the biggest problem, they do not go out in the park and understand the animal’s needs, our needs. For example, they sit in the office and discuss new plans without the need of improving the quality of life of existing species (405, Senior).

I think that transactional trust is really important, you do not say thing something and not deliver on what it is that you are promised. And it’s amazing how infrequently that happens – actually delivering on a promise. It’s really important that management is alongside people but in lot of the organisations there is there is unfortunately a division between those on the work floor, and those doing marketing and managing financial spreadsheets, etc. (370, Other - director).

Increasing *Indjobsat* through workload reduction, adequate, and meaningful provisions at all levels iseimportant but seems lacking for many. These factors may explain why *self-efficacy* was not a significant mediator between *provisions* and *Indjobsat*. This suggests a misalignment for employees, including all processes which assess how *provisions* are perceived and addressed. This is supported by our findings of the weak relationship between *processes* and levels of *Indjobsat* and *Orgjobsat*, even if more *provisions* correlate with higher *Orgjobsat*, especially with high levels of *self-efficacy*.

### 4.2 Self-efficacy and learned helplessness

Key findings show employees somewhat agree to perceiving good *individual* and *team* engagement, and mediation analyses found *self-efficacy* was a significant mediator between *team* engagement and *Orgjobsat* and *life satisfaction*. As one interviewee stated, “*Actually, this is the first place where I’ve had veterinarians that listen, you’re not just someone who’s shovelling poop all day, you actually understand the animals, which is very nice*” (130, Senior). A strong positive relationship between mean *individual* engagement and *life satisfaction* was found, and *self-efficacy* mediated the relationship between *team* engagement and *Indjobsat*. Unlike our previous finding that experience contributes to Orgjobsat and life satisfaction (86), *experience* was neither a mediator nor a moderator for these variables, and *self-efficacy* played a central role. This suggests that belief in one’s capability to overcome challenges through self-efficacy, as suggested by Bandura (31), facilitates motivation, performance, and collaboration regardless of time in the field.

This central role of Self-efficacy plays is supporious literature ed. bt prev central ro in many ways. High self-efficacy benefits individuals (31), and promoting it is important (34) because of its connection to life satisfaction (112). In everyday situations, individuals must rely on their sense of competence to evaluate difficulties. Those with strong self-belief see goals as more attainable (113), potentially affecting job satisfaction and life satisfaction. Research indicates that the effects of self-efficacy are not always positive (114, 115), with factors like perceived proximity from colleagues and organisation, including rules and shared beliefs, influencing outcomes (116, 117). For instance, employees can score high on individual job satisfaction while experiencing learned helplessness (LH), where individuals believe they are powerless after repeated failures (42). LH affects employee wellbeing through resignation to the status quo, routine limitation, and passive behaviours (8, 39, 43, 44, 118, 119). Employees may focus on small, manageable wins while avoiding larger issues where they feel powerless (120).

Our findings that *self-efficacy* was not a significant mediator between *provisions* and *Indjobsat*, along with findings that managers observe in their staff reduced engagement, problem-solving capacity, creativity, and ability to see opportunities, lower energy, neglect of wellbeing needs and increased absences, point to potential LH in ZOAQ employees.

Learned helplessness was not directly assessed using a validated instrument in this study. Rather, a pattern of convergent evidence is identified: quantitative findings from the manager observation scale, self-efficacy mediation analyses, and the financial data, combined with qualitative accounts from interviews, collectively point toward responses consistent with learned helplessness as theoretically characterised by Seligman and Maier (42) and its organisational operationalisation by Martinko and Gardner (39). This convergent approach is an appropriate and recognised method for identifying constructs not directly measured (121), and we present these findings explicitly as indicators warranting future direct measurement. Understanding how individuals develop LH and how organisational culture affects this mindset is crucial, as it negatively impacts the individual employee, and influences how well shared objectives like enhancing animal wellbeing can be achieved. Interviewees stated:

It wasn’t a good culture, it was very much a culture of fear, and people would be terrified to report mistakes, afraid of being unfairly punished. If you do anything wrong, you can be fired at any moment. And it created a very, very toxic workplace… and you had people who would bond over resisting the administration, us against the institution (119, Senior).

I raised it at upper management, and unfairness of it, and so not just for myself but also my team, so they are aware of it. Their inaction is actually quite defining. So yeah, it can get quite demoralizing. (161, Senior).

Saxena and Shah (122) found that LH is influenced by philosophical foundations, value systems, and organisational ethos, and that organisational culture significantly affects the creation or elimination of LH. LH can persist after conditions change (123), and leadership plays a crucial role in facilitating shared decision-making, role alignment, psychological safety, and communication, which are essential to reverse and support an engaged work community (124, 125). LH can affect anyone, including middle managers when organisations undergo changes (126), or those at the top. In one interview, a CEO expressed, “I am not sure if the board is truly capable to fully understand what we do, and make good decisions, particularly in developing next steps. I’d be lying if I said it is going to be ok. I mean, nobody ever does what they say” (302, CEO).

Linking to other goals, such as caring for family and saving for retirement, but facing monthly challenges in achieving these due to insufficient support, time, and funds, can Boddez et al. (127) show how failure in one life domain can affect other domains, leading to psychological suffering, including burnout and fatigue.

Pethe and Chaudhari (128) found a negative correlation between role efficacy through influence (ability to make decisions and affect changes) and LH, a negative relationship between helping relationship-receiving support from the organisation and environment and LH, and a negative relationship between confrontation and LH, indicating that people with LH view their environment as uncontrollable and avoid problems.

The patterns observed here bear comparison with Michailidis and Cropley's (129) embitterment model, in which sustained perceived injustice leads through bitterness and perseverative thinking to health damage, with overcontrolling supervision identified as a key moderator. In the present study, the pathway appears to proceed differently: through helplessness and disengagement rather than resentment and rumination. Both trajectories, however, share an upstream cause, the sustained experience of uncontrollable organisational conditions in a context of high moral commitment. These may represent two distinct but co-occurring responses to the same structural conditions, a question that warrants future research with direct LH measurement and comparative designs.

Both learned helplessness and the embitterment response (129) describe psychological costs of sustained organisational injustice in moral commitment contexts. They may represent not competing explanations but two trajectories from the same upstream cause, the structural experience of inefficacy within caring organisations, co-occurring in the same population and potentially reinforcing one another over time. Disentangling these pathways is a priority for future research in ZOAQs and related caring professions.

The implications of these patterns extend beyond individual wellbeing. Professionals who have learned, through repeated organisational experience, that their observations, concerns, and efforts produce no meaningful response will, over time, stop bringing them. The downstream consequences for animal welfare, institutional learning, and conservation effectiveness are therefore significant and represent an underexplored area of One Welfare research (69, 85, 87).

These findings make self-efficacy and LH complex subjects warranting more research in ZOAQs, including how to maintain high self-efficacy and how different aspects prevent and eliminate LH, including organisational culture.

Our findings indicate that a holistic approach is essential. Team and organisational commitment to collective goals through processes (*RHOrg*) and the feeling that one’s contributions (*RHInd* and *individual*) and needs are fulfilled appear necessary for job satisfaction (through finances and opportunities), contributing to life satisfaction. The interconnected nature is well articulated in the One Welfare approach, as one interviewee noted, “I think empathy is very important. You’re working with animals, but to not care for the people around you is such a bizarre disjunction that just can’t work” (370, other—director). The systemic approach using Bronfenbrenner’s ecological systems theory (55), echoing the One Welfare approach where animal, human, and environmental sustainability intersect (69), aligns with the all-stakeholder Rhinelands approach (66).

### 4.3 The One Care model

**Figure 7:**
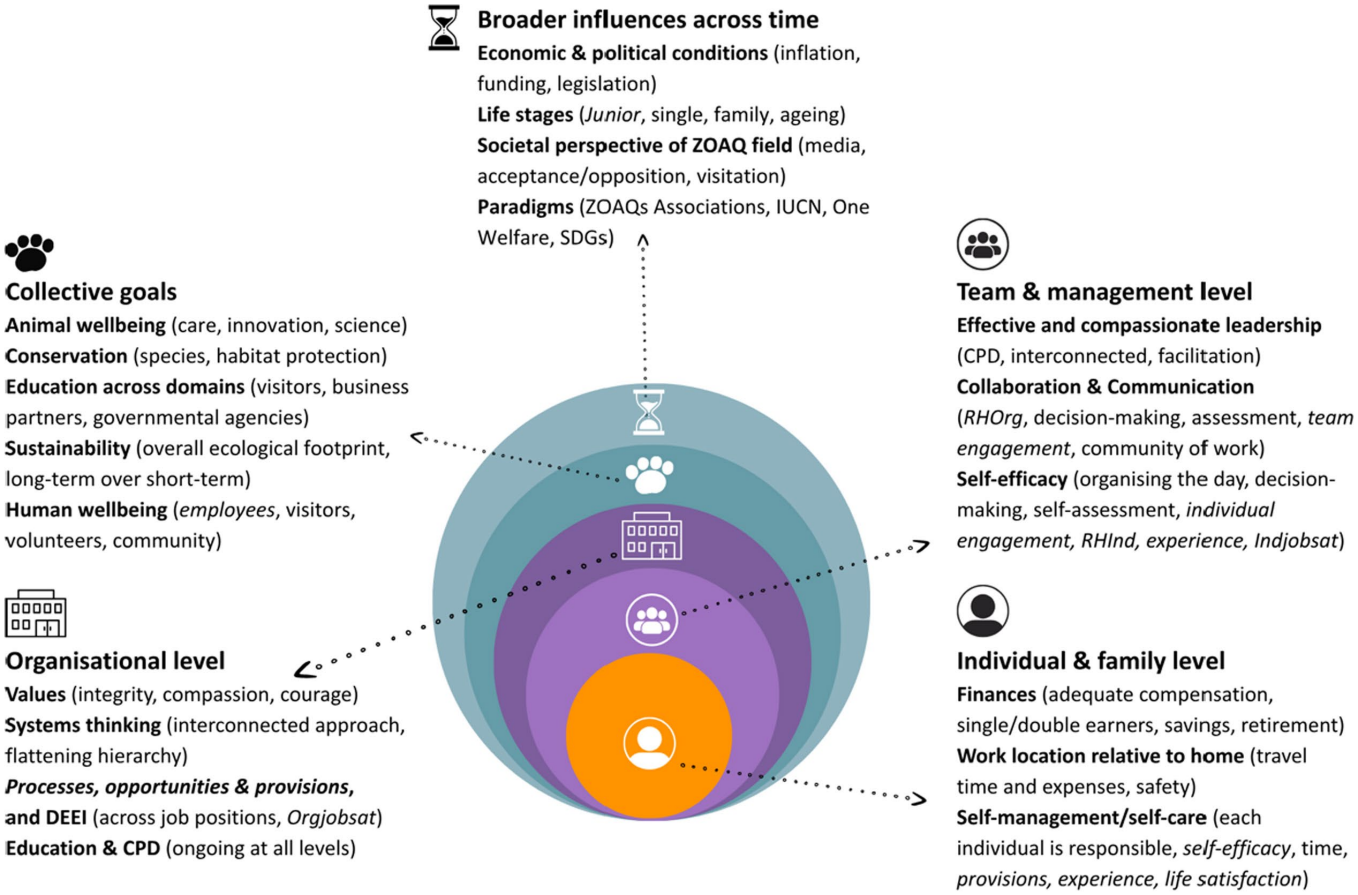
A systems approach of one care in action to promoting good wellbeing.

**Figure 8:**
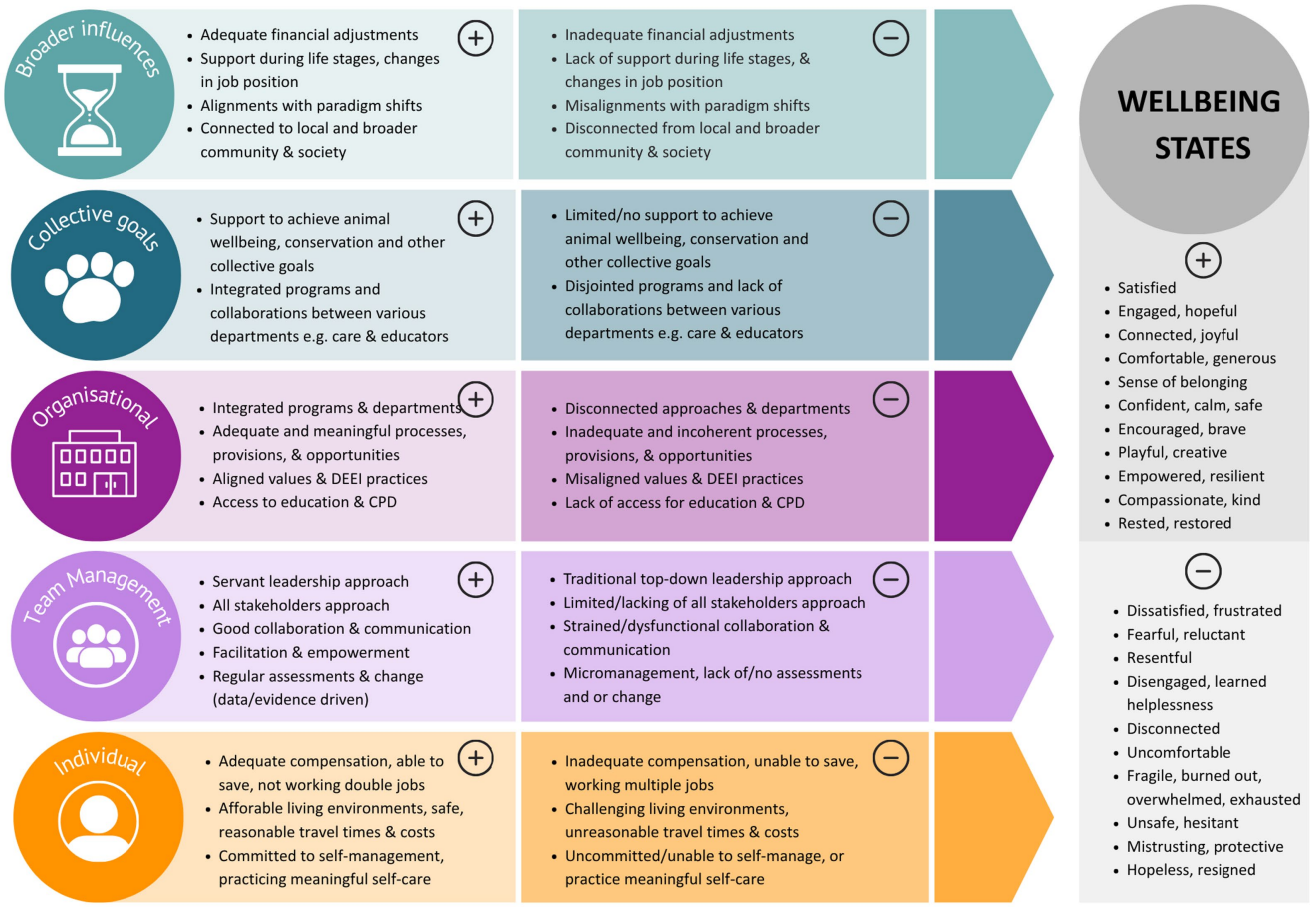
Possible impacts and outcomes, ranging from positive to negative, on employee wellbeing states.

Inspired by the BronfenbrennerEST framework, we propose a new model— One Care—in which we situate and action our findings within the and identify individual, team, management, organisational, and collective goals articulated in contemporary ZOAQs today (Figure 7). It includes inputs from our qualitative and quantitative research on aspects that influence employee wellbeing, including examples of influences not yet researched, such as broader influences across time, the impact of different collective goals, and individual aspects at and away from work. In Figure 8, we have attempted to reflect some of the possible impacts and outcomes, ranging from positive to negative, on employee wellbeing depending on the quality, adequacy, and alignments between input parameters such as processes, provisions, and organisational structures and human-based parameters such as feeling empowered or safe or feelings of LH.

## 5 Strengths and limitations

This is the first empirical study to examine employee wellbeing across multiple roles and organisational levels in ZOAQs using an integrated theoretical framework. The mixed-methods design—survey (*n* = 442) and interviews (*n* = 39) across 23 organisations in seven countries, provides both statistical rigour and qualitative depth, with five pre-specified hypotheses tested using validated scales, mediation, moderation, and PCA analyses. The proposed One Care model represents an original theoretical contribution grounding ZOAQ employee wellbeing within a systems framework with practical implications.

Limitations include: the cross-sectional design, which precludes causal inference—findings identify areas of concern and opportunity rather than proven prescriptions, and longitudinal research is needed to confirm directionality; restriction to English-language, accredited, mid-to-large ZOAQs, limiting generalisability; learned helplessness inferred rather than directly measured, with direct measurement a priority for future research; sample concentration in frontline roles by design, with CEO underrepresentation limiting the organisational-level perspective; social desirability effects, mitigated by anonymisation and the direction of findings; holistic wellbeing processes investigated at managerial level only; and the self-efficacy measure capturing perceived rather than actual competence.

## 6 Implications for practice

The present findings suggest a number of actionable directions for ZOAQ leadership and People/HV departments. These should be understood as evidence-informed recommendations, not prescriptions derived from causal evidence. Organisations might consider:

*Stop*: Continuing professional development practices that are hierarchically restricted (e.g., conferences, team building, and recognition awards predominantly accessible to management); one-size-fits-all provisions that do not account for role-specific needs; the tolerance of provision disparities across job roles without data-driven audit; the implementation of wellbeing processes without follow-up verification that they are known, understood, and experienced as meaningful by the staff they target; and processes that exist on paper but are not implemented, followed up, or understood at the working level.

*Start*: Mapping provision equity across all job roles, with particular attention to frontline caregivers; implementing self-efficacy-supportive management practices including coaching access, psychological safety, and meaningful participation in decision-making; establishing regular points of contact on the workfloor, and meaningful regular structured consultations between management and all staff levels with documented follow-up; coaching access for all staff (not only managers) as a tool for building self-efficacy.

*Measure differently*: Organisations should measure provision equity by role (not just aggregate satisfaction), the proportion of staff with adequate CPD access, and the degree to which processes (*holprocesses*) are meaningful, known, and understood by the staff they are meant to protect. Move beyond satisfaction scores as the primary wellbeing indicator and measure diverse indicators; and systematic tracking of manager-observed LH indicators, including reduced initiative, withdrawal from problem-solving, and passive compliance, as early warning signals, as well as boredom, or work-related rumination.

## 7 Conclusion

A people-centred culture creates environments with supportive leadership, effective communication, and promotes help-seeking behaviours and adaptable structures and processes which support collective goals such as animal wellbeing and conservation, while simultaneously considering the needs and wellbeing of employees. Empowering staff by reducing and eliminating managerial control, increasing leadership support and facilitation, and increasing individual and collective decision-making supports collaboration, and promotes self-efficacy and team commitment. Adopting a stakeholder approach like Rhinelands can improve and support individual and team engagement as drivers towards collective goals and flatten the hierarchy towards a community of work in which insights from the work floor drive facilitation from supporting departments such as those in managerial positions and in the People Department (formerly known as HR). A systems approach - one that holds animal, human, and environmental wellbeing as interdependent rather than competing concerns, offers the most coherent framework for understanding and improving life in ZOAQs. This study contributes one empirical layer to that framework; the architecture of a genuinely flourishing field will require many more.

## Data Availability

The raw data supporting the conclusions of this article will be made available by the authors, without undue reservation.

## References

[1] Brando S Rachinas-Lopes P Goulart VDLR Hart LA. Understanding job satisfaction and occupational stressors of distinctive roles in zoos and aquariums. Animals. (2023) 13:2018. doi: 10.3390/ani13122018, 37370528 PMC10295341

[2] Bunderson JS Thompson JA. The call of the wild: zookeepers, callings, and the double-edged sword of deeply meaningful work. Admin Sci Q. (2009) 54:32–57. doi: 10.2189/asqu.2009.54.1.32

[3] Maddock A. (2023). Relationships between Stress, Burnout, Mental Health and Well-Being in Social Workers. Available online at: https://academic.oup.com/bjsw/article/54/2/668/7379806 (Accessed April 23, 2026).

[4] Madigan DJ Kim LE Glandorf HL Kavanagh O. Teacher burnout and physical health: a systematic review. Int J Educ Res. (2023) 119:102173. doi: 10.1016/j.ijer.2023.102173

[5] Roșca AC Mateizer A Dan C-I Demerouti E. Job demands and exhaustion in firefighters: the moderating role of work meaning. A cross-sectional study. Int J Environ Res Public Health. (2021) 18:18. doi: 10.3390/ijerph18189819, 34574742 PMC8472436

[6] Søvold L. E. Naslund J. A. Kousoulis A. A. Saxena S. Qoronfleh M. W. Grobler C. . (2021). *Prioritizing the Mental Health and Well-Being of Healthcare Workers: An Urgent Global Public Health Priority*. Available online at: https://www.frontiersin.org/journals/public-health/articles/10.3389/fpubh.2021.679397/full (Accessed April 23, 2026). 10.3389/fpubh.2021.679397 PMC8137852 34026720

[7] Bridgeman PJ Bridgeman MB Barone J. Burnout syndrome among healthcare professionals. Am J Health Syst Pharm. (2018) 75:147–152. doi: 10.2146/ajhp170460 29183877

[8] Rizvi YS Sikand R. Learned helplessness at the workplace and its impact on work involvement: an empirical analysis. Glob Bus Rev. (2020) 2020:0972150920976693. doi: 10.1177/0972150920976693

[9] Sorensen L. C. Ladd H. F. (2020). *The Hidden Costs of Teacher Turnover*. Available online at: https://journals.sagepub.com/doi/full/10.1177/2332858420905812 (Accessed April 23, 2026).

[10] Westover JH. The Hidden Costs of Neglecting Employee Well-Being: A Financial Case for Prioritizing Burnout Prevention. Orem, Utah: HCI Consulting (2024).

[11] Bautista T. Roman G. Khan M. Lee M. Sahbaz S. . (2023). What is well-being? A scoping review of the conceptual and operational definitions of occupational well-being. Available online at: https://pubmed.ncbi.nlm.nih.gov/38028344/ (Accessed April 23, 2026). 10.1017/cts.2023.648 PMC10643923 38028344

[12] Jarden A Roache A. What Is Wellbeing? Int J Environ Res Public Health. (2023) 20:5006. doi: 10.3390/ijerph20065006 36981914 PMC10049282

[13] Michaelson J Mahony S Schifferes J. Measuring Wellbeing: A Guide for Practitioners. London, United Kingdom: New Economics Foundation (2012).

[14] Anker M Anker R. Anker Methodology. New York: Anker Research Institute (2025).

[15] Perrott A. Improving wellbeing through a modern, integrated experience. He Rourou. (2022) 2:1. doi: 10.54474/herourou.v2i1.7145

[16] Ray TK. Work related well-being is associated with individual subjective well-being. Ind Health. (2022) 60:242–52. doi: 10.2486/indhealth.2021-0122, 34732595 PMC9171114

[17] AL Mutairy HM AL Drgham NM AL Masoud AS AL Shammari FH AL Wohabe RA Abdullah EAA . ACHIEVING BALANCE: a review of the integration of personal life and work in healthcare field. Int J Med Health Sci. (2018) 4:4. doi: 10.53555/eijmhs.v4i4.232

[18] Batool Z Hasan H Sajid GM. Does job-satisfaction cause life-satisfaction? New evidence using Lewbel methodology. Pak Dev Rev. (2020) 59:357–76. doi: 10.30541/v59i3pp.357-376

[19] American Psychological Association. (2018) APA Dictionary of Psychology Life Satisfaction. Available online at: https://dictionary.apa.org/ (Accessed April 23, 2026).

[20] Keyes CLM Shmotkin D Ryff CD. Optimizing well-being: the empirical encounter of two traditions. J Pers Soc Psychol. (2002) 82:1007–22. doi: 10.1037/0022-3514.82.6.1007 12051575

[21] Ring L Höfer S McGee H Hickey A O’Boyle CA. Individual quality of life: can it be accounted for by psychological or subjective well-being? Soc Indic Res. (2007) 82:443–61. doi: 10.1007/s11205-006-9041-y

[22] Diener E Emmons RA Larsen RJ Griffin S. The satisfaction with life scale. J Pers Assess. (1985) 49:71. doi: 10.1207/s15327752jpa4901_13, 16367493

[23] Diener E Suh EM Lucas RE Smith HL. Subjective well-being: three decades of progress. Psychol Bull. (1999) 125:276–302. doi: 10.1037/0033-2909.125.2.276

[24] Fredrickson BL. Positivity: Groundbreaking Research Reveals How to Embrace the Hidden Strength of Positive Emotions, Overcome Negativity, and Thrive. New York: Crown Publishers/Random House (2009).

[25] Warr P. A study of psychological well-being. Br J Psychol. (1978) 69:111–21. doi: 10.1111/j.2044-8295.1978.tb01638.x, 626801

[26] Montuori P Sorrentino M Sarnacchiaro P Di Duca F Nardo A Ferrante B . Job satisfaction: knowledge, attitudes, and practices analysis in a well-educated population. Int J Environ Res Public Health. (2022) 19:14214. doi: 10.3390/ijerph192114214, 36361094 PMC9656398

[27] Locke A. E. (1976). The nature and causes of job satisfaction. *Handbook of Industrial and Organizational Psychology*. Available online at: https://cir.nii.ac.jp/crid/1573105975115113600 (Accessed April 23, 2026).

[28] Judge TA Zhang S Glerum DR. "Job satisfaction". In: Essentials of Job Attitudes and Other Workplace Psychological Constructs. London and New York: Routledge (2020).

[29] Dreer B. Teachers’ well-being and job satisfaction: the important role of positive emotions in the workplace. Educ Stud. (2024) 50:61–77. doi: 10.1080/03055698.2021.1940872

[30] Velez MJ Marujo HA Charepe Z Querido A Laranjeira C. Well-being and dispositional hope in a sample of Portuguese citizens: the mediating role of mental health. Eur J Investig Health Psychol Educ. (2024) 14:7. doi: 10.3390/ejihpe14070140, 39056655 PMC11275276

[31] Bandura A. "Self-efficacy". In: Ramachaudran VS, editor. Encyclopedia of human Behavior, vol. 4. San Diego: Academic Press (1994). p. 71–81.

[32] Bandura A. (2017). *Self Efficacy*. Available online at: https://albertbandura.com/albert-bandura-self-efficacy.html (Accessed April 23, 2026).

[33] Meng Q. Chinese university teachers’ job and life satisfaction: examining the roles of basic psychological needs satisfaction and self-efficacy. J Gen Psychol. (2022) 149:327–48. doi: 10.1080/00221309.2020.1853503 33269661

[34] Cayupe JC Bernedo-Moreira DH Morales-García WC Alcaraz FL Peña KBC Saintila J . Self-efficacy, organizational commitment, workload as predictors of life satisfaction in elementary school teachers: the mediating role of job satisfaction. Front Psychol. (2023) 14:1066321. doi: 10.3389/fpsyg.2023.1066321, 37325744 PMC10264599

[35] Kuşcu Karatepe H Tiryaki Şen H Türkmen E. Predicting work performance and life satisfaction of nurses and physicians: the mediating role of social capital on self-efficacy and psychological resilience. Perspect Psychiatr Care. (2022) 58:2542–51. doi: 10.1111/ppc.13092 35430728

[36] Topino E Svicher A Di Fabio A Gori A. Satisfaction with life in workers: a chained mediation model investigating the roles of resilience, career adaptability, self-efficacy, and years of education. Front Psychol. (2022) 13:1093. doi: 10.3389/fpsyg.2022.1011093, 36211910 PMC9539406

[37] Hassan S Gull M Farasat M Asif R. Servant leadership and employee well-being in sustainable organizations: a self-efficacy perspective. J Excellence Manag Sci. (2023) 2:2

[38] Hiroto DS Seligman ME. Generality of learned helplessness in man. J Pers Soc Psychol. (1975) 31:311–27. doi: 10.1037/h0076270

[39] Martinko MJ Gardner WL. Learned helplessness: An alternative explanation for performance deficits. Acad Manag Rev. (1982) 7:195–204. doi: 10.5465/amr.1982.4285559

[40] Abramson LY Seligman ME Teasdale JD. Learned helplessness in humans: critique and reformulation. J Abnorm Psychol. (1978) 87:49–74. doi: 10.1037/0021-843X.87.1.49, 649856

[41] Maier SF Seligman MEP. Learned helplessness at fifty: insights from neuroscience. Psychol Rev. (2016) 123:349–67. doi: 10.1037/rev0000033 27337390 PMC4920136

[42] Seligman ME Maier SF. Failure to escape traumatic shock. J Exp Psychol. (1967) 74:1–9. doi: 10.1037/h0024514 6032570

[43] Andrieu CFA Milhabet I Denis-Noël A Steiner DD. Voice in the void: from voice to acquiescent silence over time as learned helplessness in organizations. J Work Organ Psychol. (2024) 40:103–18. doi: 10.5093/jwop2024a9

[44] Balakrishnan S. (1990). *Learned Helplessness in Organizations*. Available online at: https://vslir.iima.ac.in:8443/jspui/handle/11718/352 (Accessed April 23, 2026).

[45] Tayfur O. The antecedents and consequences of learned helplessness in work life. Inf Manag Bus Rev. (2012) 4:417–27. doi: 10.22610/imbr.v4i7.996

[46] Ji T Loh MY De Jonge J Peeters MCW Taris TW Dollard MF. “Are you feeling safe?” An investigation of psychosocial safety climate in the relations of job characteristics and employee exhaustion and engagement. Ind Health. (2025) 63:3–13. doi: 10.2486/indhealth.2024-0027 38763741 PMC11779518

[47] Brando S. A Culture of Care: Individual, Leadership, and Organisational Aspects of Human Wellbeing in Zoos and Aquariums.Zoo and Aquarium Association (2022a).

[48] Brando S. (2021) ‘System Failures’ in the zoo and Aquarium Related Domains, Rebooting to see the Broader picture & ACT for more Positive Changes. Part 1: An Overview (No. 56) [Broadcast]. Available online at: https://www.animalconcepts.eu/podcasts/interbeing-by-animalconcepts/episodes/2147573432 (Accessed April 23, 2026).

[49] Elkington J. "The triple bottom line". In: Environmental Management: Readings and Cases SAGE (2008)

[50] Miller K. (2020). The Triple Bottom Line: What It Is & Why It’s Important. Business Insights Blog. Available online at: https://online.hbs.edu/blog/post/what-is-the-triple-bottom-line (Accessed April 23, 2026).

[51] Brando S. (2022) Good news and Actions: Peoples and Organisations as Forces for good. Available online at: https://www.animalconcepts.eu/blog/good-news-and-actions-peoples-and-organisations-as-forces-for-good-by-sabrina-brando (Accessed April 23, 2026).

[52] Brando S. (2023) InterBeing. Available online at: https://www.animalconcepts.eu/blog/INTERBEING (Accessed April 23, 2026).

[53] Brando S Jenkins H Rachinas-Lopes P Tamayo Moreno M Norman M Verhoeven I. Doing the right Things and doing Things right: Action Speak Louder than Words.Zoo Aquarium Association (ZAA) (2024).

[54] Bronfenbrenner U. Ecology of childhood. School Psychol Rev (world). (1980) 9:568. doi: 10.1080/02796015.1980.12086568

[55] Bronfenbrenner U. Ecological systems theory. Encycl Psychol. (2000) 3:129–33. doi: 10.1037/10518-046

[56] Crawford M. Ecological systems theory: exploring the development of the theoretical framework as conceived by bronfenbrenner. J. Public Health Issues Pract. (2020) 4:17. doi: 10.33790/jphip1100170

[57] Hermosa-Rodríguez AM. Intervenciones en estrés laboral: Un análisis a partir del modelo bioecológico de Bronfenbrenner. Psicol Salud. (2019) 29:2. doi: 10.25009/pys.v29i2.2583

[58] Tong P An IS. Review of studies applying Bronfenbrenner’s bioecological theory in international and intercultural education research. Front Psychol. (2024) 14:1233925. doi: 10.3389/fpsyg.2023.1233925, 38259539 PMC10801006

[59] Zhang Y. F. (2018) Using Bronfenbrenner’s Ecological Approach to Understand Academic Advising with International Community College Students. Available online at: https://ojed.org/jis/article/view/230

[60] Bone KD. The bioecological model: applications in holistic workplace well-being management. Int J Workplace Health Manag. (2015) 8:256–71. doi: 10.1108/IJWHM-04-2014-0010

[61] Tharikh SM Hamzah SR. Application of the wellbeing theory on air traffic controllers: a model on how to flourish in practice at the workplace. Int J Acad Res Bus Soc Sci. (2020) 10:522–32. doi: 10.6007/ijarbss/v10-i5/7225

[62] Wang F Huang R Lim WM Zhang J. Perceived employability of international doctoral students in the UK: applying Bronfenbrenner’s ecological systems theory. Stud High Educ. (2024) 50:2327–45. doi: 10.1080/03075079.2024.2412833

[63] Eriksson M Ghazinour M Hammarström A. Different uses of Bronfenbrenner’s ecological theory in public mental health research: what is their value for guiding public mental health policy and practice? Soc Theory Health. (2018) 16:414–33. doi: 10.1057/s41285-018-0065-6

[64] Renn K. A. Smith B. R. G. (2023) Ecological Models in Higher Education Research: Overview and Synthesis. Available online at: https://onlinelibrary.wiley.com/doi/10.1002/he.20491 (Accessed April 23, 2026).

[65] Anderson J Boyle C Deppeler J. The Ecology of Inclusive Education.Brill (2014).

[66] Peters J. (2021) *Rijnlands organiseren* Managementboek.nl Available online at: https://www.managementboek.nl/boek/9789024439126/rijnlands-organiseren-jaap-peters (Accessed April 23, 2026).

[67] Albert M. Capitalisme contre capitalisme. Paris, France: FeniXX (1991). Available online at: https://brill.com/display/book/9789462096929/BP000004.xml

[68] Peters J Weggeman M. The Rhineland Way: Reintroducing a European style of Organisation. Amsterdam, The Netherlands: Uitgeverij Business Contact (2010).

[69] García Pinillos R. Appleby M. C. Manteca X. Scott-Park F. Smith C. Velarde A. (2016) One Welfare – a Platform for Improving human and animal Welfare. Available online at: https://bvajournals.onlinelibrary.wiley.com/doi/abs/10.1136/vr.i5470 10.1136/vr.i5470 27770094

[70] Garcia Pinillos R. (2022) One Welfare: A Framework to Improve animal Welfare and human well-Being *CABI*. Available online at: https://www.cabidigitallibrary.org/doi/book/10.1079/9781786393845.0000 (Accessed April 23, 2026).

[71] Olmos-Vega FM Stalmeijer RE Varpio L Kahlke R. A practical guide to reflexivity in qualitative research: AMEE guide no. 149. Med Teach. (2023) 45:241–51. doi: 10.1080/0142159X.2022.2057287 35389310

[72] Braun V Clarke V. Thematic Analysis. London, United Kingdom: SAGE Publications Ltd (2021a).

[73] Byrne D. A worked example of Braun and Clarke’s approach to reflexive thematic analysis. Qual Quant. (2022) 56:1391–412. doi: 10.1007/s11135-021-01182-y

[74] Pavan AD O’Quin J Roberts ME Freed CL. Using a staff survey to customize burnout and compassion fatigue mitigation recommendations in a lab animal facility. J Am Assoc Lab Anim Sci. (2020) 59:139–47. doi: 10.30802/AALAS-JAALAS-19-000080, 32024579 PMC7073397

[75] Marton B. Kilbane T. Nelson-Becker H. (2020). Exploring the loss and disenfranchised grief of animal care workers. Available online at: https://www.tandfonline.com/doi/abs/10.1080/07481187.2018.1519610 (Accessed April 23, 2026). 10.1080/07481187.2018.1519610 30654733

[76] Moore IC Coe JB Adams CL Conlon PD Sargeant JM. The role of veterinary team effectiveness in job satisfaction and burnout in companion animal veterinary clinics. J Am Vet Med Assoc. (2014) 245:513–24. doi: 10.2460/javma.245.5.513, 25148093

[77] Lincoln Y. S. Guba E. G. (1985). *Naturalistic Inquiry—Yvonna S. Lincoln, Egon G. Guba—Google Books*. Available online at: https://books.google.es/books?hl=en&lr=&id=2oA9aWlNeooC&oi=fnd&pg=PA7&ots=0vnwPdQexl&sig=dVELCh9FIZYGnKL5uydTo7zzoI0&redir_esc=y#v=onepage&q&f=false (Accessed April 23, 2026).

[78] Braun V Clarke V. To saturate or not to saturate? Questioning data saturation as a useful concept for thematic analysis and sample-size rationales. Qual Res Sport Exerc Health. (2021) 13:201–16. doi: 10.1080/2159676X.2019.1704846

[79] Clarke V. Hayfield N. Moller N. Tischner I. (2017) Once upon a Time (Chapter 3)—Collecting Qualitative Data. Cambridge, United Kingdom.

[80] Boivin GP Markert RJ. Factors affecting the vocational calling of laboratory animal care and research employees. J Am Assoc Lab Anim Sci. (2016) 55:769–74. 27931315 PMC5113878

[81] Townshend K. (2023). Satisfaction with life scale (SWLS). In Handbook of Assessment in Mindfulness Research. Available online at: https://link.springer.com/referenceworkentry/10.1007/978-3-030-77644-2_83-1#DOI

[82] PROMIS® Health Organization (2008) PROMIS Item Bank v.1.0—General Self-Efficacy. (Accessed April 23, 2026).

[83] Kupst MJ Butt Z Stoney CM Griffith JW Salsman JM Folkman S . Assessment of stress and self-efficacy for the NIH toolbox for neurological and behavioral function. Anxiety Stress Coping. (2015) 28:531–44. doi: 10.1080/10615806.2014.994204 25577948 PMC4515370

[84] Dolbier CL Webster JA McCalister KT Wallon MW Steinhardt MA. Reliability and validity of a single-item measure of job satisfaction. Am J Health Promot. (2005) 19:194–8. doi: 10.4278/0890-1171-19.3.194 15693347

[85] Bacon H Vigors B Shaw DJ Waran N Dwyer CM Bell C. Zookeepers – the most important animal in the zoo? J Appl Anim Welf Sci. (2023) 26:634–46. doi: 10.1080/10888705.2021.2012784 34894904

[86] Brando S Donisete Lima Rodrigues Goulart V Buchanan-Smith H Rey Planellas S Caes L. The role of self-care in perceptions of satisfaction with life, organisational job satisfaction, and self-efficacy in zoo and aquarium professionals. Front Vet Sci. (2025) 12:1677195. doi: 10.3389/fvets.2025.1677195 41427147 PMC12716087

[87] Cole J Fraser D. Zoo animal welfare: the human dimension. J Appl Anim Welf Sci. (2018) 21:49–58. doi: 10.1080/10888705.2018.1513839, 30325229

[88] Barbieri B Bellini D Scaratti G Mondo M Pinna R Galletta M . Examining the interplay between positive and negative bureaucracy characteristics and job satisfaction: the moderating role of resistance to change for neo-managerial approaches. Acad Q. (2024) 29:16–34. doi: 10.54337/academicquarter.i29.9900

[89] Hossli N Natter M Algesheimer R. On the importance of congruence between personal and work values – how value incongruence affects job satisfaction: a multiple mediation model. Int J Wellbeing. (2024) 14:2905. doi: 10.5502/ijw.v14i3.2905

[90] Antonsen S Almklov P Fenstad J. Reducing the gap between procedures and practice—lessons from a successful safety intervention. Saf Sci Monit. (2008) 12:1–16.

[91] Weldon D White A Bouldin A Gregory D Kuo GM Fuentes D. Organizational values: essential substrate for professional identity formation. Am J Pharm Educ. (2023) 87:542. doi: 10.1016/j.ajpe.2023.100542, 37419703

[92] Singh K. Leadership role in aligning values and strategic operations. Int J Multidiscip Res. (2025) 7:113. doi: 10.36948/ijfmr.2025.v07i04.40113

[93] Steenkamp PL Basson JS. A meaningful workplace: framework, space and context. HTS Teol Stud. (2013) 69:9 (Selected). doi: 10.4102/hts.v69i1.1258

[94] Banu SS Wahid K Khan HD. A study on determinants of employee turnover in the workplace environment. Int J Res Appl Sci Eng Technol. (2025) 13:2121–8. doi: 10.22214/ijraset.2025.72615

[95] Sethumadhavan R. (2025). Creating a positive workplace for the employee wellbeing [chapter]. IGI Global Scientific Publishing. (creating-a-positive-workplace-for-the-employee-wellbeing). Available online at: https://services.igi-global.com/resolvedoi/resolve.aspx (Accessed April 23, 2026).

[96] Pandey D Ganatra NJ. Does biasedness starts with HR? - analysis and outcomes and suggestions for transparent practices. J Manag Res Anal. (2025) 11:185–8. doi: 10.18231/j.jmra.2024.031

[97] Mubango H Muchowe R Mataruka L. Bridging the gap: navigating human resources management bias to promote equality and inclusivity in the workplace. Int J Res Innov Soc Sci. (2025) 25:1559–69.

[98] Murtaza G Roques O Siegrist J Talpur Q-A. Unfairness and stress—an examination of two alternative models: organizational-justice and effort–reward imbalance. Int J Public Adm. (2023) 46:602–12. doi: 10.1080/01900692.2021.2009854

[99] Virtanen M Elovainio M. Justice at the workplace: a review. Cambridge Q Healthc Ethics. (2018) 27:306–15. doi: 10.1017/S0963180117000639 29509127

[100] Gesiarz F De Neve J-E Sharot T. The Motivational Cost of Inequality: Pay Gaps Reduce the Willingness to Pursue Rewards (SSRN Scholarly Paper No. 3484543).Social Science Research Network (2019). Available online at: https://pmc.ncbi.nlm.nih.gov/articles/PMC7473543/

[101] Rutte CG Messick DM. An integrated model of perceived unfairness in organizations. Soc Justice Res. (1995) 8:239–61. doi: 10.1007/BF02334810

[102] Muralidhar L. B. Sathyanarayana N. Swapna H. R. Hukkeri S. V. Reshma Sultana P. H. Muralidhar L. B. . (2025). Machine learning advancing diversity equity and inclusion in data-driven HR practices [Chapter]. IGI Global Scientific Publishing. (machine-learning-advancing-diversity-equity-and-inclusion-in-data-driven-hr-practices). Available online at: https://services.igi-global.com/resolvedoi/resolve.aspx (Accessed April 23, 2026).

[103] Tariq M. U. (2025). Harnessing technology to combat Bias: innovative tools and techniques in HR [chapter]. IGI Global Scientific Publishing. (harnessing-technology-to-combat-bias). Available online at: Https://Services.Igi-Global.Com/Resolvedoi/Resolve.Aspx?Doi=10.4018/979-8-3693-7132-9.Ch006 (Accessed April 23, 2026).

[104] Cusumano C Warmath D. Mind the gap: investigating how financial well-being shapes job satisfaction through burnout. J Workplace Behav Health. (2024) 41:–203. doi: 10.1080/15555240.2024.2441208

[105] Li J Kaltiainen J Hakanen JJ. Overbenefitting, underbenefitting, and balanced: different effort–reward profiles and their relationship with employee well-being, mental health, and job attitudes among young employees. Front Psychol. (2023) 14:1020494. doi: 10.3389/fpsyg.2023.1020494 37051602 PMC10083407

[106] Shah SKA Ali Z Tariq M. The impact of unfair compensation on employee morale, job satisfaction, and performance: a quantitative analysis in Pakistan’s health sector. Global Manage Sci Rev. (2024) 3:65–76. doi: 10.31703/gmsr.2024(IX-III).06

[107] Roslinda JA Andias AB. Exploring employee motivation: a comprehensive analysis of key motivational factors across diverse industries. Int J Multidiscip. (2025) 6:2985–3005. doi: 10.11594/ijmaber.06.06.28

[108] Andrews CG. Comparative Analysis of Management and Employee job Satisfaction and Policy Perceptions [Doctor of Philosophy. Denton, Texas: University of North Texas (2003).

[109] Wei W Ayo EB. Enhancing organizational effectiveness: a study on human resource strategies, employee productivity, and turnover in SMEs. Int J Innov Sci Res Technol. (2024) 24:769–82. doi: 10.38124/ijisrt/IJISRT24JUL621

[110] Dong B. A systematic review of the organizational culture change literature and future outlook. Front Hum Soc Sci. (2023) 3:118–24. doi: 10.54691/fhss.v3i4.4783

[111] Murphy K. Assessment of employee well-being on organisational effectiveness & productivity: a literature review. Int J Bus Manag. (2024) 19:p26. doi: 10.5539/ijbm.v19n3p26

[112] Guo Z. Exploring the effects of happiness motives and general efficacy on life satisfaction. SHS Web of Conferences. (2025) 222:03012. doi: 10.1051/shsconf/202522203012

[113] Thorne TN Milyavskaya M Werner K Leduc-Cummings I Saunders B Inzlicht M. The Personal Goal Difficulty - Progress Paradox: Unraveling the Role of Self-Efficacy on Perceptions of Goal Difficulty (SSRN Scholarly Paper No. 4651737).Social Science Research Network (2023). Available online at: https://papers.ssrn.com/sol3/papers.cfm?abstract_id=4651737

[114] Bachrach DG Rapp TL Rapp AA Ogilvie J. “Too much” self-efficacy? Understanding the curvilinear consequences of between-person self-efficacy through a moderated-mediation model of perceived proximity and employee effort. Group Org Manag. (2023) 48:1544–81. doi: 10.1177/10596011211070098

[115] Flammer A. Nakamura Y. (2002). An den Grenzen der Kontrolle. In Selbstwirksamkeit und Motivationsprozesse in Bildungsinstitutionen. pedocs. Available online at: http://nbn-resolving.de/urn:nbn:de:0111-opus-39322 (Accessed April 23, 2026).

[116] Harush R Lisak A Glikson E. The bright side of social categorization: the role of global identity in reducing relational conflict in multicultural distributed teams. Cross Cult Strateg Manag. (2017) 25:134–56. doi: 10.1108/CCSM-11-2016-0202

[117] Ruiller C Van Der Heijden B Chedotel F Dumas M. “You have got a friend”: the value of perceived proximity for teleworking success in dispersed teams. Team Perform Manag. (2018) 25:2–29. doi: 10.1108/TPM-11-2017-0069

[118] Kankus R Cavalier R. Combating organizationally induced helplessness. Qulaity Progress. (1995) 28:89.

[119] Yüksel F Ibrahim YA Ozyurt SS. Learned helplessness in public administration: the case of san Diego City. Mediterr J Soc Sci. (2015). doi: 10.5901/mjss.2015.v6n2p151

[120] Heath N. (2023) Learned Helplessness at work: What it is and how to beat it. INTHEBLACK. Available online at: https://intheblack.cpaaustralia.com.au/work-life/learned-helplessness-at-work-what-and-how-to-beat-it (Accessed April 23, 2026).

[121] Creswell JW Plano Clark VL. Designing and Conducting Mixed Methods Research. 3rd ed. Thousand Oaks, CA: SAGE (2018).

[122] Saxena S Shah H. Effect of organizational culture on creating learned helplessness attributions in R&D professionals: a canonical correlation analysis. Vikalpa. (2008) 33:25–46. doi: 10.1177/0256090920080203

[123] Jordan A. C. (2025). *Learned Helplessness at Work: What Leaders Can Do | Psychology Today*. Available online at: https://www.psychologytoday.com/us/blog/leading-with-connection/202506/learned-helplessness-at-work-what-leaders-can-do (Accessed April 23, 2026).

[124] Ashforth BE. The organizationally induced helplessness syndrome: a preliminary model. Canad J Admin Sci. (1990) 7:30–6. doi: 10.1111/j.1936-4490.1990.tb00532.x

[125] Kaabomeir N Mazhari K Arshadi N Karami M. How supervisors can support employees’ needs and motivation? An experimental study based on SDT. Curr Psychol. (2023) 42:17206–18. doi: 10.1007/s12144-022-02922-5 PMC8902267 35283610

[126] Silvet J. Learned helplessness during organisational change. Ann Alexandru Ioan Cuza University Econ. (2013) 60:93–103. doi: 10.2478/aicue-2013-0028

[127] Boddez Y Van Dessel P De Houwer J. Learned helplessness and its relevance for psychological suffering: a new perspective illustrated with attachment problems, burn-out, and fatigue complaints. Cognit Emot. (2022) 36:1027–36. doi: 10.1080/02699931.2022.2118239 36107793

[128] Pethe S Chaudhari S. Role efficacy dimensions as correlates of occupational self efficacy and learned helplessness. Indian J Ind Relat. (2000) 35:507–18.

[129] Michailidis E Cropley M. Exploring predictors and consequences of embitterment in the workplace. Ergonomics. (2017) 60:1197–206. doi: 10.1080/00140139.2016.1255783 27801614

[130] Hayes AF. Introduction to mediation, moderation, and conditional process analysis: a regression-based approach (3rd ed.). Guilford Press. (2022).

