# Supplementary material for: Raising the bar: a systems approach to promoting good wellbeing and self-efficacy in zoo and aquarium professionals
Source: Front Vet Sci. 2026 Jul 9;13:1746998. doi: 10.3389/fvets.2026.1746998 (PMC13393462; doi:10.3389/fvets.2026.1746998)
Supplement: Supplementary file 1 [file Data_Sheet_1.DOCX]

**Supplementary Materials**

**Survey and Interview Questions**

*"Raising The Bar: The Key Role of a Systems Approach to Promoting Good Wellbeing and Improve Self-Efficacy in Zoo and Aquarium Professionals"*

Brando, Buchanan-Smith, Rey Planellas & Caes | Frontiers in Veterinary Science | MS 1746998

# **Appendix A — Survey Questions**

Table A1 presents all survey questions used in the analyses reported in this manuscript. Table A2 presents additional survey questions collected as part of the broader PhD project but not analysed in this paper.

| **Q No.** | **Question** | **Scale / Response Options** | **Type / Reference** |
| --- | --- | --- | --- |
| **ALL PARTICIPANTS** | | | |
| **DEMOGRAPHICS** | | | |
| **Q2** | My job position is (please select 1) | Junior animal caregiver / Senior animal caregiver / Curator / Veterinarian / Veterinary professional e.g., veterinary nurse / Animal welfare scientist/coordinator / CEO / Other e.g., nutritionists / Prefer not to say / Optional: Comments | *Demographics (Brando et al., 2023)* |
| **Q3** | Please identify your gender (please select 1) | Female / Male / Nonbinary / Prefer not to disclose / Optional: Comments | *Demographics (Spiel et al., 2019)* |
| **Q4** | Please indicate your age using the age ranges (please select 1) | 21-25 / 26-30 / 31-35 / 36-40 / 41-45 / 46-50 / 51-55 / 56-60 / 61-65 / 66+ / Prefer not to say / Optional: Comments | *Demographics* |
| **Q5** | I have worked in the animal care and welfare domain for (please select 1) | Less than a year / 1-5 years / 6-10 years / 11-15 years / 16-20 years / 21-25 years / More than 25 years / Prefer not to say / Optional: Comments | *Demographics (Brando et al., 2023)* |
| **Q6** | Please select your highest education level (please select 1) | On the job trained / High school / Higher education certification/vocational / BSc / MSc / PhD / Other / Prefer not to say / Optional: Comments | *Demographics* |
| **Q7** | I work (please select 1) | Part-time (up to 20 hours per week) / Part-time (more than 20 hours per week) / Full-time / Prefer not to say | *Demographics* |
| **Q8** | When at work (please select 1) | I only work the hours I am paid for / I sometimes work additional hours that I am not paid for (e.g., I sometimes work through breaks, or stay late) / I often work additional hours that I am not paid for / Prefer not to say | *Demographics* |
| **Q9** | Financially I can make it through a month (please select 1) | Never (i.e., my income is not nearly enough to cover my basic expenses) / Mostly (i.e., my income covers most of my basic expenses, but sometimes it is a bit short) / Completely (i.e., my income always covers my basic expenses but there is little left for anything else) / Comfortably (i.e., all basic expenses are covered and some of my income can be spent on wants rather than needs) | *Demographics* |
| **Q10** | I receive adequate financial compensation for the work that I do | Likert scale: Strongly disagree / Disagree / Somewhat disagree / Neither agree nor disagree / Somewhat agree / Agree / Strongly agree / Prefer not to say / Optional: Comments | *Demographics* |
| **Q11** | Opportunities for development - Continuing Professional Development (CPD). Please select all that apply | I have continued on the job training / I have satisfactory opportunities for CPD / My employer pays all costs required for me to engage in CPD / My employer pays some of the costs required for me to engage in CPD / I can use my work days to attend an event CPD / I have access to online resources and courses / I have no access to continued professional development / Prefer not to say | *Demographics* |
| **Q12** | Please share any other opportunities if you wish | Comment box | *Demographics* |
| **Q13** | I consider my current job to be (please select 1) | A way to make money only; I would be just as happy doing other work / A job that I have some interest in doing / A career in which I look to remain in the field / Primarily a calling; it's my passion / Prefer not to say / Optional: Comments | *Boivin & Markert (2016)* |
| **Q15** | Does your current position include supervision of other employees? (please select 1) | Yes / No / Prefer not to say / Comments |  |
| **TEAM ENGAGEMENT** | | | |
| **Q16** | Team engagement. My co-workers have respect for me / My co-workers trust me / My co-workers come to work with a good attitude / When a co-worker is experiencing a personal crisis, everyone pulls together to ensure things still run smoothly / There are complementing personalities on the team / In our organisation people are open to change / My manager checks in regularly on team members / My manager is compassionate / My manager helps us work through disagreements / My manager cares about their own wellbeing | Likert scale: Strongly disagree / Disagree / Somewhat disagree / Neither agree nor disagree / Somewhat agree / Agree / Strongly agree / Prefer not to say / Optional: Comments | *Moore et al. (2014)* |
| **Q17** | Individual engagement. I am encouraged to expand my knowledge to enhance my role / I am recognised as an important part of the team / I have a voice in the decisions made in the organisation / I am comfortable speaking up if I have a problem / I lack confidence in the abilities of some of my co-workers | Likert scale: Strongly disagree / Disagree / Somewhat disagree / Neither agree nor disagree / Somewhat agree / Agree / Strongly agree / Prefer not to say / Optional: Comments | *Moore et al. (2014)* |
| **ORGANISATIONAL JOB SATISFACTION** | | | |
| **Q18** | Organisational job satisfaction. The organisation appreciates any extra effort from me / The organisation listens to complaints from me / The organisation really cares about my wellbeing / The organisation is willing to help me when I need a special favour / The organisation takes pride in my accomplishments at work | Likert scale: Strongly disagree / Disagree / Somewhat disagree / Neither agree nor disagree / Somewhat agree / Agree / Strongly agree / Prefer not to say / Optional: Comments | *Boivin & Markert (2016)* |
| **RHINELANDS INDIVIDUAL (RHInd)** | | | |
| **Q19** | Organisational approach (Rhinelands Individual). I am proud of the work I can do / I feel I have enough time to do my work well / I feel I have the space to do right by my job / I feel supported by my organisation / I feel management trusts me / I know that intelligent disobedience is allowed in my organisation (do what you know is right even if it goes against what you are asked) | Likert scale: Strongly disagree / Disagree / Somewhat disagree / Neither agree nor disagree / Somewhat agree / Agree / Strongly agree / Prefer not to say / Optional: Comments | *Peters (2021)* |
| **PROVISIONS** | | | |
| **Q20** | My organisation provides (please select all that apply) | Appropriate clothing / Adequate equipment / Lunch / Tea and coffee / Opportunities to participate in volunteering programs of choice / Team bonding exercises or events / Opportunities for awards of recognition for exceptional work / Access to health care / Access to a coach or trainer (e.g., when a skill needs to be mastered) / Self-care training / Access to a counsellor (e.g., when a beloved animal passes away) / A whistle-blower policy / Prefer not to say |  |
| **WORK-LIFE BALANCE** | | | |
| **Q21** | I think (please select 1) | My work-life and personal-life integration is balanced more towards work / My work-life and personal-life integration is balanced more towards life / My work-life and personal-life integration is mostly even |  |
| **Q22** | I think (please select 1) | My work-life and personal-life integration is problematic / My work-life and personal-life integration is satisfactory / My work-life and personal-life integration is harmonious |  |
| **INDIVIDUAL JOB SATISFACTION** | | | |
| **Q23** | I am satisfied with my job (single item) | Likert scale: Strongly disagree / Disagree / Somewhat disagree / Neither agree nor disagree / Somewhat agree / Agree / Strongly agree / Prefer not to say / Optional: Comments | *Dolbier et al. (2005)* |
| **Q24** | Do you see yourself as being in a helping profession? | Yes / No / Prefer not to say. (A helping profession is defined as 'a profession that nurtures the growth of, or addresses the problems of, an individual's physical, psychological, cognitive, or emotional well-being.') |  |
| **Q25** | My facility has a union | Yes / No / I don't know / Prefer not to say / Comment |  |
| **Q26** | I am part of a union | Yes / No / I don't know / Prefer not to say / Comment |  |
| **Q27** | I feel that I contribute and make a difference in: Animal care & wellbeing / Education / Conservation / Research / Engagement | Likert scale: Strongly disagree / Disagree / Somewhat disagree / Neither agree nor disagree / Somewhat agree / Agree / Strongly agree / Prefer not to say / Optional: Comments |  |
| **SELF-CARE (5 DOMAINS)** | | | |
| **Q35** | Physical self-care. In the last month I... Ate regularly (e.g., breakfast, lunch, and dinner) / Ate foods that promote energy and well-being (e.g., fruits & vegetables) / Exercised consistently (3-4 times a week) / Got 7-9 hours of sleep per night / Took time off when I got sick / Took vacations / Made time for physical rest / Made time for relaxation / Went for regular medical check-ups / Digitally unplugged 1 hour before sleep | 6-point Likert: Never thought about that / Never / Rarely / Sometimes / Often / Always / Prefer not to say | *Butler (2010); Saakvitne & Pearlman (1996)* |
| **Q36** | Intellectual self-care. In the last month I... Read outside of work requirements / Tried new things / Made time for personal development / Did something creative (e.g., painted, cooked, or baked something new) / Improved my ability to say no when I want to / Said no to people crossing my boundaries / Wore clothes I like | 6-point Likert: Never thought about that / Never / Rarely / Sometimes / Often / Always / Prefer not to say | *Butler (2010); Saakvitne & Pearlman (1996)* |
| **Q37** | Emotional self-care. In the last month I... Allowed for quality time with others who have a positive impact on my life / Re-read favourite books or re-watched favourite movies / Made time for self-reflection / Identified rewarding activities outside of work / Allowed myself to accept time, help, advice, etc. from others / Allowed myself to feel my feelings / Sought personal therapy / Identified and limited my exposure to those who were not supportive / Noticed my feelings and what provokes them / Spoke with a trusted person about something that bothered me / Expressed my feelings in a safe outlet / Attended support groups / Did something nice for myself | 6-point Likert: Never thought about that / Never / Rarely / Sometimes / Often / Always / Prefer not to say | *Butler (2010); Saakvitne & Pearlman (1996)* |
| **Q38** | Spiritual/connecting to meaning self-care. In the last month I... Spent time in nature / Have been open to inspiration / Cherished my own optimism and hope / Was aware of nonmaterial aspects of life / Meditated / Prayed / Participated in a community that shares my core values / Contributed to causes in which I believe / Read inspirational literature / Listened to inspiring music | 6-point Likert: Never thought about that / Never / Rarely / Sometimes / Often / Always / Prefer not to say | *Butler (2010); Saakvitne & Pearlman (1996)* |
| **Q39** | Workplace professional self-care. In the last month I... Allowed for breaks during the day / Took time to connect with co-workers / Took quiet space to complete tasks / Set limits/boundaries as needed / Made sure my workspace was comfortable / Participated in projects or tasks that were exciting and rewarding / Worked with a manager or co-worker to balance workload / Negotiated needs (benefits, bonuses, raise, etc.) as required | 6-point Likert: Never thought about that / Never / Rarely / Sometimes / Often / Always / Prefer not to say | *Butler (2010); Saakvitne & Pearlman (1996)* |
| **GENERAL SELF-EFFICACY** | | | |
| **Q40** | General Self-Efficacy. I can manage to solve difficult problems if I try hard enough / If someone opposes me, I can find the means and ways to get what I want / It is easy for me to stick to my aims and accomplish my goals / I am confident that I could deal efficiently with unexpected events / Thanks to my resourcefulness, I know how to handle unforeseen situations / I can solve most problems if I invest the necessary effort / I can remain calm when facing difficulties because I can rely on my coping abilities / When I am confronted with a problem, I can usually find several solutions / If I am in trouble, I can usually think of a solution / I can usually handle whatever comes my way | 5-point Likert: I am not at all confident / I am rarely confident / I am sometimes confident / I am often confident / I am very confident / Prefer not to say | *PROMIS® Item Bank v1.0 (2020)* |
| **SATISFACTION WITH LIFE** | | | |
| **Q41** | Satisfaction with Life Scale. In most ways my life is close to my ideal / The conditions of my life are excellent / I am satisfied with my life / So far I have gotten the important things I want in life / If I could live my life over, I would change almost nothing | 5-point Likert (adapted from 7-point): Strongly disagree / Somewhat disagree / Neither agree nor disagree / Somewhat agree / Strongly agree / Prefer not to say | *Diener et al. (1985)* |
| **MANAGERS ONLY** | | | |
| **Q45** | I have worked in this managerial position for (please select 1) | Less than a year / 1-5 years / 6-10 years / 11-15 years / 16-20 years / 21-25 years / More than 25 years / Prefer not to say / Comments |  |
| **Q46** | I have worked in this facility for (please select 1) | Less than a year / 1-5 years / 6-10 years / 11-15 years / 16-20 years / 21-25 years / More than 25 years / Prefer not to say / Comments |  |
| **Q47** | I started at this facility in the position I am in now (please select 1) | Yes / No, I moved up from another position in this facility / No, I came from a similar position in another facility / No, I moved up from another position in another facility / Prefer not to say / Comments |  |
| **Q48** | Over my career I have worked in number of parks. Please indicate number of organisations | Comment box |  |
| **Q49** | Over my career I have worked in number of countries | Comment box |  |
| **Q50** | As I moved up the ladder to a leadership position, I have completed people management courses (please select 1) | Yes. Please list the types of courses or degrees / No / Prefer not to say |  |
| **Q51** | I manage a team of (please select 1) | 1-5 people / 6-10 people / 11-15 people / 16 or more / Prefer not to say |  |
| **Q52** | In the organisation there are other managers in a position like mine (please select 1) | No, I am the only one / Yes — 1-5 people / 6-10 people / 11-15 people / 16-20 people / 21 or more people / Prefer not to say |  |
| **MANAGER PERCEPTIONS — WORKING WITH TEAM AND MANAGEMENT** | | | |
| **Q53** | In my position as a manager, I feel supported by the team(s) I supervise e.g., good communication, getting the job done together | Likert scale: Strongly disagree / Disagree / Somewhat disagree / Neither agree nor disagree / Somewhat agree / Agree / Strongly agree / Prefer not to say / Optional: Comments |  |
| **Q54** | In my position as a manager, I feel supported by upper management e.g., clear goals, budget | Likert scale: Strongly disagree / Disagree / Somewhat disagree / Neither agree nor disagree / Somewhat agree / Agree / Strongly agree / Prefer not to say / Optional: Comments |  |
| **Q55** | In my position as a manager, I feel trapped between the team(s) I supervise and upper management (e.g., conflicting goals, poor communication) | Likert scale: Strongly disagree / Disagree / Somewhat disagree / Neither agree nor disagree / Somewhat agree / Agree / Strongly agree / Prefer not to say |  |
| **Q56** | In my position as a manager, I feel supported by other managers in the organisation (e.g., for advice, exchange) | Likert scale: Strongly disagree / Disagree / Somewhat disagree / Neither agree nor disagree / Somewhat agree / Agree / Strongly agree / Prefer not to say |  |
| **Q57** | In my position as a manager, I think I have a clear understanding of the needs of the team(s) I supervise (e.g., resources needed, animal needs) | Likert scale: Strongly disagree / Disagree / Somewhat disagree / Neither agree nor disagree / Somewhat agree / Agree / Strongly agree / Prefer not to say |  |
| **MANAGER OBSERVATIONS — LEARNED HELPLESSNESS INDICATORS** | | | |
| **Q58** | In my position as a manager, I observe in the team I supervise: Social isolation or withdrawal, refusing offers of help / Neglecting personal wellbeing needs / Increased absence from work / Coming to work when ill / Lack of engagement / Reduced energy / Reduced capacity to problem solve / Reduced creativity / Reduced desire to work with people / Passivity / Emotional numbness / Reduced communication / Decreased ability to see opportunities / Avoidance of interactions with management / General withdrawal from life outside work | Likert scale: Strongly disagree / Disagree / Somewhat disagree / Neither agree nor disagree / Somewhat agree / Agree / Strongly agree / Prefer not to say |  |
| **HOLISTIC WELLBEING PROCESSES (ISO 45001/45003)** | | | |
| **Q59** | Is the organisation ISO 45001:2018 - Occupational health and safety management systems certified? (please select 1) | Yes / No / I don't know / Prefer not to say / Comment |  |
| **Q60** | Is the organisation ISO 45003:2021 - Occupational health and safety management — Psychological health and safety at work certified? (please select 1) | Yes / No / I don't know / Prefer not to say / Comment |  |
| **Q61** | The organisation has a written human wellbeing charter; stipulating details of the philosophy, ethics, and practical conduct of promoting good human wellbeing (please select 1) | Yes / No / I don't know / Prefer not to say / Comment with examples |  |
| **Q62** | The organisation has a written animal welfare charter; stipulating details of the philosophy, ethics, and practical conduct of promoting good animal welfare (please select 1) | Yes / No / I don't know / Prefer not to say / Comment with examples |  |
| **Q63** | The organisation has a good understanding of the needs and expectations of the staff regarding: financial security / social interaction and support / diversity and inclusion / recognition, reward and accomplishment / personal development and growth / equal opportunity and fair treatment at work | Likert scale: Strongly disagree / Disagree / Somewhat disagree / Neither agree nor disagree / Somewhat agree / Agree / Strongly agree / Prefer not to say |  |
| **Q64** | The organisation provides opportunities for continued personal and professional development (please check all that apply) | Days off to attend event / Fully paid conferences/events and days off to attend / Fully paid conferences / First aid / People management skills / Communication skills / Human wellbeing courses such as occupational health and safety management, psychological health and safety / Other / Prefer not to say |  |
| **Q65** | The organisation consults with workers at regular intervals about their position and work including specific aspects affecting human wellbeing such as job satisfaction, work conditions and continued development (please select 1) | Yearly / Quarterly / Other, please specify / I don't know / Prefer not to say |  |
| **Q66** | The organisation has a whistle-blower policy (please select 1) | Yes / No / In the process of development / I don't know / Prefer not to say |  |
| **Q67** | The organisation determines the risks and opportunities that need to be addressed: psychosocial hazards / prevention of injury and ill-health / strategies for workers returning to work / opportunities for improvement, including promotion of wellbeing at work / the development, review and maintenance of occupational health and safety | Likert scale: Strongly disagree / Somewhat disagree / Neither agree nor disagree / Somewhat agree / Strongly agree / Prefer not to say | *ISO 45003* |
| **Q69** | When communicating, the organisation: demonstrates top management commitment / provides opportunities for feedback from workers / outlines the importance of effective occupational health and safety management / promotes a culture that supports the intended outcomes of the occupational health and safety management system | Likert scale: Strongly disagree / Somewhat disagree / Neither agree nor disagree / Somewhat agree / Strongly agree / Prefer not to say | *ISO 45003* |
| **Q71** | To promote health, safety and well-being at work and manage psychosocial risk in the event of an emergency, the organisation has: recognised that a wide range of emergency situations can impact psychological health / provided guidance and resources to workers and managers / taken action to manage the psychosocial risk of emergency events | Likert scale: Strongly disagree / Somewhat disagree / Neither agree nor disagree / Somewhat agree / Strongly agree / Prefer not to say | *ISO 45003* |
| **Q73** | For appropriate rehabilitation and return-to-work programmes, the organisation has: access to occupational health services / provided access to confidential debriefing / provided information about rehabilitation and return-to-work options | Likert scale: Strongly disagree / Somewhat disagree / Neither agree nor disagree / Somewhat agree / Strongly agree / Prefer not to say | *ISO 45003* |
| **Q75** | The organisation: conducts internal audits at planned intervals including consideration of psychosocial risks / uses the findings of audits to assess the effectiveness of management of psychosocial risks / identifies gaps in performance to identify opportunities to continually improve | Likert scale: Strongly disagree / Somewhat disagree / Neither agree nor disagree / Somewhat agree / Strongly agree / Prefer not to say | *ISO 45003* |
| **RHINELANDS ORGANISATIONAL (RHOrg)** | | | |
| **Q77** | In relation to your organisation (please rate): Craftsmanship is at the heart of our business / Hierarchy does not mean much to us / The dominant leadership style is top-down / Professionals and teams of professionals are self-managing / Measurable objectives play an important role for us / Sustainability and corporate social responsibility are serious strategic issues for us / Our top managers steer more towards the short term than the long term / There is a lot of consultation because we think consensus is important / Managers understand the expertise on the work floor / Ultimately, the organisation is there for the people in the organisation, and the people are not there for the organisation / We work with corporate targets and staff will be judged on them / We work in a very results-oriented way / Who's in charge gets the final say, instead of who knows (the answer), gets to the final say / Customers are ultimately more important than employees / We are based on trust, an agreement is an agreement. Planning, control, and management systems are therefore secondary / The individual performance, that's what matters | Likert scale: Strongly disagree / Somewhat disagree / Neither agree nor disagree / Somewhat agree / Strongly agree / Prefer not to say | *Peters (2021)* |
| **SURVEY QUESTIONS NOT USED IN THESIS ANALYSES (Table A2)** | | | |
| **Q14** | What are the predominant taxa with which you currently work? (please select all that apply) | Mammals (primates, elephants, marine mammals) / Mammals (hoof stock, bears, big cats) / Mammals (all others) / Birds / Reptiles / Fishes / Amphibians / Invertebrates / Not working directly with animals at present / Prefer not to say | *Demographics* |
| **Q28** | I see/experience predominantly for animals in our care: who are well most of the time / who exhibit trust in me and other caregivers / traumatised (e.g., animals who are scared most of the time, have high levels of anxiety) / who spend a lot of time relaxing, playing and in positive states / who spend a lot of time in negative states (e.g., stereotypies, self-harm) | Likert scale |  |
| **Q29** | Tools we currently use to understand how the animals are faring are (please select all that apply) | Regular health checks / Preventive health care/check / Behavioural observations / Paper recording keeping / Animal care and welfare monitoring programs (e.g., ZIMS, formal welfare monitoring) / Other / Prefer not to say |  |
| **Q30** | When an animal that we are attached to dies or has to be euthanised, the organisation provides: an opportunity to say goodbye / more information to know what has happened to the animal / an opportunity to have a ceremony open for all to attend / a special place where we can go to celebrate and remember the animal | Please select all that apply |  |
| **Q31** | If at our organisation we have to euthanise an animal: The veterinarian takes the decision alone / The veterinarian and senior animal team members make the decision together / The whole animal team is part of the decision-making process / All are informed of the decision, including those on a day off / Other, please specify | Please select all that apply |  |
| **Q32** | With regards to the end of an animal's life, I feel that: an animal that I have had an attachment to should have a remembrance ceremony / there should be an opportunity for me to talk about an animal's death / it is acceptable that animals are disposed of in a bin / it is acceptable that animals are used as food / it is acceptable that animals are used for scientific research | Likert scale |  |
| **Q33** | Feel welcome to add any comments regarding caring for animals, end of life, and when animals die | Comment box |  |
| **Q34** | Please let us know what would help you to do your work properly (e.g., any support strategies that can be put in place and how)? | Comment box |  |

# **Appendix B — Survey Demographic Questions**

Table B1 presents the demographic questions included in the survey, covering job position, gender, age, years working in the field, highest educational level, job type, working hours, financial situation, financial compensation, CPD opportunities, job calling, supervision responsibilities, union membership, and contribution to collective goals.

*Note: All demographic questions are included within Table A1 above (Q2–Q15, Q21–Q27). A separate demographic summary table (frequencies and distributions) is available upon request from the corresponding author.*

# **Appendix C — Interview Questions**

Table C1 presents all interview questions used in the qualitative component of this study. Semi-structured interviews were conducted online via Microsoft Teams between 26 October 2023 and 30 January 2024 with 39 participants (20 male, 19 female) who had completed the survey and volunteered for follow-up. Interviews lasted approximately one hour. All interviews were recorded, transcribed, and anonymised.

## **Opening and Introduction**

Welcome! Thank you for making time in your day to speak to me. I look forward to chatting with you today.

My name is Sabrina and I'm conducting this interview in my role as a PhD researcher at the University of Stirling, Scotland, and the director of AnimalConcepts, a consultancy company helping individuals and organisations care for animals, the planet, and themselves. I have been in the animal field for over 30 years working across the globe. I am a psychologist with an MSc in Animal Studies and am interested in the human-animal interface.

Thank you for having signed and returned the consent form so that I can record this session for future analysis.

This conversation is confidential and the data will be completely anonymous. The recording will be stored securely, and only me and my supervisors will have access.

If you would like to take a short break during the conversation or stop the conversation altogether, just let me know.

Do you have any questions before we get started?

## **Broad Opening Questions**

**1.** Share with me your motivation for joining the interview process?

**2.** Describe your role and approach to your daily work?

*Possible prompts / deeper dives:*

— What do you like the most at your job?

— Share how you feel supported in your job by your organisation.

*Possible prompt: How does your organisation help to meet your needs and/or preferences so you can do your work and deliver the desired outcomes?*

— What are the most reoccurring issues in your organisation that are not being solved for you?

— If you were the director at the Zoo, and hence you had the capacity to change things, what would be your top 3 changes you would focus on?

*For CEOs: What are your three top changes you would like to focus on if there were no financial or time restrictions (i.e., their ideal situation)?*

## **Zoom In Questions**

**3.** Research in caring professions shows there can be benefits and challenges for the carer as well as the one being cared for — in your case the animals in your care. Could you please share some examples of how your wellbeing (poor or good) related to the animal's wellbeing directly?

**4.** Share what you think are the important steps forward that need to happen to support the wellbeing of the animals in your direct care and/or across the organisation?

**5.** Share how you feel supported at work to care for your own wellbeing?

*Potential follow-ups:*

— Does this support extend to caring for your own wellbeing in your personal life?

— Please share some examples from work/team/home.

— Perhaps elaborate on what could be improved?

## **Team and Leadership Questions**

**6.** When it comes to working in your team, as well as with leadership on a regular basis, share how you feel supported at work with respect to your own wellbeing as well as the animals' wellbeing?

*Potential follow-ups:*

— Please share some examples from work/team.

— Perhaps elaborate on what could be improved?

**7.** Share how you feel about the organisation as a whole, and how it is doing in the space of human wellbeing in the workplace?

*Potential follow-ups:*

— Please share some examples from work/team.

— Perhaps elaborate on what could be improved?

## **Closing Question**

**8.** To conclude this conversation, can you share a success story of how a difficult situation was handled well? For example, with respect to the wellbeing of an animal, a combined team effort, or anything else that comes to mind.

*Possible prompt: Think of an example of another zoo if needed.*

*— End of Supplementary Materials —*
